# Transcriptomic and metabolomic analyses reveal the mechanism of color difference between two kinds of *Cistanche deserticola* before and after drying

**DOI:** 10.3389/fpls.2024.1506523

**Published:** 2025-01-23

**Authors:** Tiantian Zhu, Jing Zhang, Tianle Liu, Shuai Zhang, Baimei Yang, Li Xu, Lei Zhao, Mengfei Li, Ling Jin

**Affiliations:** ^1^ College of Pharmacy, Gansu University of Traditional Chinese Medicine, Lanzhou, China; ^2^ Northwest Collaborative Innovation Center for Traditional Chinese Medicine Co-constructed by Gansu Province & Ministry of Education (MOE) of People's Republic of China (PRC), Lanzhou, China; ^3^ Engineering Research Center for Evaluation, Protection, and Utilization of Rare Traditional Chinese Medicine Resources, Lanzhou, China; ^4^ Provincial-Level Key Laboratory for Chinese Tibet Herbal Chemicals and Quality Research in Gansu Colleges and Universities, Lanzhou, China; ^5^ State Key Laboratory of Aridland Crop Science, Gansu Agricultural University, Lanzhou, China

**Keywords:** *Cistanche deserticola*, color, flavonoids, iridoids, metabolomic, transcriptome

## Abstract

**Introduction:**

Cistanche deserticola is an important traditional Chinese herbal medicine. The fresh cistanche squamous stem is typically yellow-white and brown after drying. Oil cistanche is a cistanche variant with a purple squamous stem that turns black after drying. The color difference between oil cistanche and cistanche is obvious, and the former has a higher market price. However, the mechanism underlying the color difference of oil cistanche and cistanche remains unclear.

**Methods:**

This study evaluated the total flavone contents in oil cistanche and cistanche and compared the differential metabolites and differentially expressed genes (DEGs) and the contents of iridoid of dried oil cistanche and cistanche samples were determined by high-performance liquid chromatography, and finally the polysaccharides contents of them were determined to comprehensively analyze the formation mechanism of color difference between oil cistanche and cistanche.

**Results:**

The results showed that the total flavonoid content in oil cistanche was significantly higher than that in cistanche. Metabolomic analysis identified 50 differentially accumulated metabolites (DAMs) (34 up-regulated and 16 down-regulated), including carbohydrates, terpenoids, and flavonoids. Moreover, 3,376 DEGs were selected, among which significant up-regulated of *IGS1* and *CYP84A1* and down-regulated of *4CLL1*, *F6H2-2-1* and *5MAT1* genes jointly regulated flavonoid biosynthesis and affected the accumulation of differentially accumulated metabolites. Significant up-regulated of the *CCD7* gene affected carotenoid component production, and significant up-regulated of the *UGT85A24* gene promoted the accumulation of geniposidic acid. In addition, the contents of iridoid and polysaccharide in oil cistanche were significantly higher than those in cistanche.

**Discussion:**

The differential expression of flavonoids and terpenoid differential metabolites and *CYP84A1*, *5MAT1*, *FLS*, *UGT85A24* and *CCD7* mainly caused the purple color of fresh oil cistanche. Dried samples of oil cistanche were darker in color than those of cistanche, due to the higher content of iridoids and polysaccharides in the former. This study preliminarily revealed the causes of the color differences between oil cistanche and cistanche, and provided references for the systematic study of cistanche and its germplasm resources, as well as for the breeding of *C. deserticola*.

## 1 Introduction

*Cistanche deserticola* (*C. deserticola*) is a fleshy stem with squamous originating from the plants *Cistanche deserticola* YC Ma or *Cistanche tubulosa* (Schenk) Wight (CPC, 2020). *C. deserticola* is a traditional and valuable Chinese herbal medicine, which has the effects of tonifying kidney Yang, nourishing essence and blood, and moistening bowel. Modern research shows that the main chemical components of *C. deserticola* are phenylethanoid glycosides, iridoids and their glycosides, lignans and their glycosides, polysaccharides, monoterpenes, organic acids, etc., which have a variety of biological activities such as anti-inflammatory, antioxidant, anti-tumor, memory improvement, bowel-loosening and intestines-moistening, and reproductive function improvement (Xue et al., 2024; Fu et al., 2018; Gai et al., 2015). In addition, *C. deserticola*, as a medicinal and edible plant, has high potential economic value. The squamous stem of fresh *C. deserticola* are usually yellow, but a type of intraspecific variation named “oil cistanche” has been discovered in the genuine producing areas of *C. deserticola*. Its squamous stem are dark purple, and after drying, its color is darker than that of *C. deserticola* (Zhang et al., 2023; Chen et al., 2008; Ma et al., 2006; Zhou et al., 2022). This variation type is evenly distributed in Inner Mongolia, Gansu, Xinjiang, and other places in China. According to market surveys, oil cistanche is more expensive than cistanche and is more favored by consumers. Current research on the medicinal material of oil Cistanche is limited to the quantification of certain active components. The color difference between the squamate stems of oil cistanche and cistanche, as well as the underlying mechanisms, remains unexplored. Understanding the causes of color variation between fresh samples and dried medicinal materials of oil cistanche and cistanche is of great significance for the study of oil cistanche.

There are many metabolites related to plant color, including polyphenols (anthocyanins, flavonols-quercetin, and curcumin), isoprenoids (iridoids, carotenoids, and quinones), alkaloids, and other compounds, which can form colors such as red, blue, and purple under specific conditions (Sigurdson et al., 2017; Brudzyńska et al., 2021). For example, purple notoginseng roots have higher flavonoid and anthocyanin contents, purple taro tubers have higher anthocyanin content than white taro tubers, the red-skinned roots of Salvia miltiorrhiza contain higher levels of tanshinones, the yellower the surface color of Anemarrhena asphodeloides, the higher the content of mangiferin and neomangiferin, and the brighter yellow color of Cortex Phellodendri Chinensis is associated with higher levels of berberine and phellodendrine compared to other colors (He et al., 2023; Wei et al., 2015; Jiang et al., 2020; Su et al., 2019). Geniposidic acid, a component of iridoids, is a natural blue pigment, reacts with amino acids such as glutamate and arginine under the action of citric acid to form purple-red polymers (Li et al., 2020). When genipin and geniposide glycosides mix with methylamine, they produce blue-black and purplish-red pigments (Su et al., 2013). The conjugated double bonds and various functional groups contained in carotenoid molecules contribute to the color range of many fruits and vegetables, spanning from yellow, red to orange (Bartley and Scolnik, 1995; Hornero-Méndez and Mínguez-Mosquera, 2000). β-Carotene has been found to combine with chlorophyll or lutein, forming chlorophyll-carotenoid complexes that absorb light in the orange or red spectrum and produce greens, purples, or blues (Wieruszewski, 2002).

Previous studies have clearly elucidated the metabolic pathways of flavonoids and terpenoids. The synthesis of flavonoids begins with the conversion of phenylalanine into 4-coumaroyl-CoA through the phenylpropanoid pathway. This is followed by the sequential action of chalcone synthase (CHS), chalcone isomerase (CHI), and either flavone synthase I or flavone synthase II to synthesize flavones. Under the influence of flavonoid 3’-hydroxylase, dihydroflavonols are formed. Dihydroflavonols are then converted into anthocyanins through the actions of dihydroflavonol 4-reductase (DFR), aureusidin synthase (ANS), glycosyltransferase (GT), and other enzymes (Tohge et al., 2017). Terpenoids include natural pigments such as carotenoids and plant colorants like iridoids. Both are synthesized from geranyl diphosphate (GPP), which is produced through the mevalonate pathway and the methylerythritol phosphate pathway. GPP can be converted into geraniol, the starting substance for the secoiridoid pathway, under the catalysis of geraniol synthase (GES). Through the catalysis of enzymes such as geraniol 10-hydroxylase, 8-hydroxygeraniol oxidoreductase, and iridoid synthase, iridoid compounds are formed (Dai et al., 2023). Carotenoids are formed from GGPP through a series of enzymatic catalysis, including phytoene synthase and phytoene desaturase, ultimately resulting in lycopene. Lycopene is then catalyzed by lycopene cyclase, lycopene β-cyclase, and lycopene ϵ-cyclase to produce β-carotene and α-carotene, respectively (Wu et al., 2024). The formation of the purple color in the scaly stems of Cistanche deserticola may be related to flavonoids, anthocyanins, and terpenoids. Furthermore, anthocyanins, as a subclass of flavonoids, confer color to plants by modifying them with sugars and acyl acids. The production of anthocyanins is stimulated by sucrose-specific signaling pathways (Tohge et al., 2017). Therefore, evaluating and analyzing the mechanism of color difference formation between oil cistanche and cistanche using terpenoid, phenylpropanoid, and polysaccharide-related synthetic pathways is of great significance for the study of cistanche.

The color of fresh Cistanche deserticola tends to darken after drying, which may be related to the decomposition of iridoids, as well as the content of polysaccharides, 5-hydroxymethylfurfural (5-HMF), and alkaloids (Liu et al., 2020). Studies have shown that the darkening of processed traditional Chinese medicinal materials such as Scrophularia ningpoensis, Rehmannia glutinosa, and ovate catalpa fruit is caused by the decomposition of iridoid components (Duan et al., 2013). As the color of Rehmannia glutinosa gradually intensifies, the content of iridoid glycosides gradually decreases, while the content of polysaccharides gradually increases (Xue et al., 2023). The darker the color of traditional Chinese medicinal materials, the higher the polysaccharide content (Xue et al., 2017).

To elucidate the reasons for the purple and yellow hues in the fresh scaly stems of oil cistanche and cistanche, respectively, as well as the darker color of dried oil cistanche compared to dried cistanche, this study employed spectrophotometry to determine the total flavonoid content in fresh samples of both species. Transcriptome and metabolome analyses were conducted on fresh samples to investigate differential metabolites and genes. Additionally, the main active components, iridoids and polysaccharides, were measured in dried medicinal materials to further explore the causes of color differences between oil cistanche and cistanche. This research aims to lay a foundation for enriching the germplasm resources of *C. deserticola*.

## 2 Materials and methods

### 2.1 Plant material

**Figure 1:**
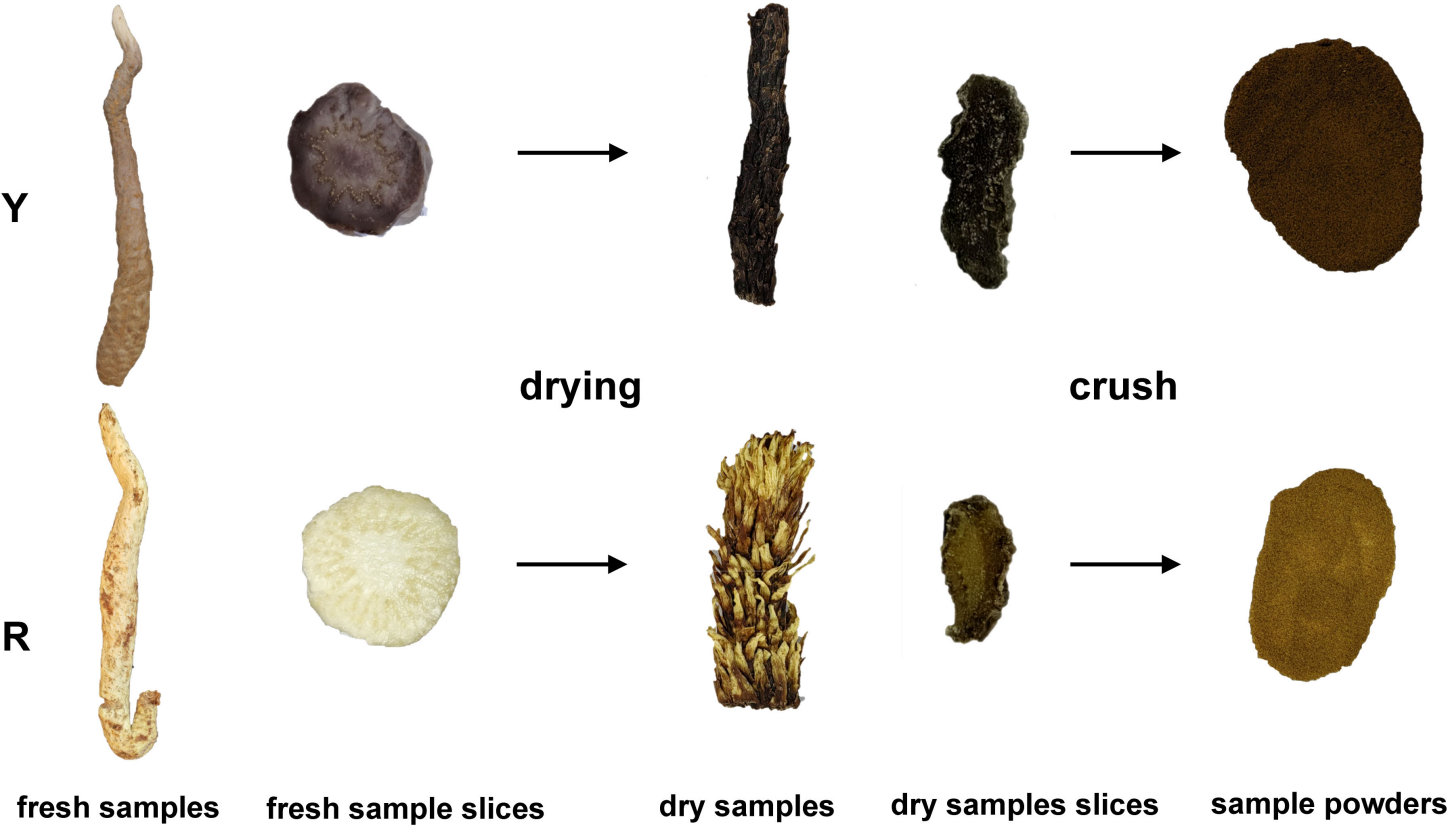
Appearance characteristics of oil cistanche (Y) and cistanche (R).

In this study, the upper stem of oil cistanche and Cistanche squamata were selected as the test material (**Figure 1**). All plants were collected from a bionic wild cultivated site (elevation 1171.5 m; 39°34′53′′N,104°48′3′′E) in Alxa East County, Inner Mongolia. During the growth stage, the plants were maintained in the environment and collected during the medicinal stage (October to November 2023). To ensure consistency between samples, the oil cistanche and cistanche samples were collected from three individuals of the same species, representing three biological replicates. Fresh squamate stems from the upper part were collected, cleaned thoroughly, stored in liquid nitrogen, and transported to the laboratory where they were preserved in a -80°C ultra-low temperature freezer. These samples were used for total flavonoid content determination and metabolomics and transcriptomics analysis. After drying, the samples were used for the determination of cyclic enol ether iridoids and polysaccharides.

### 2.2 Determination of chemical composition content

#### 2.2.1 Determination of total flavonoid contents

Fresh samples (0.5 g) were placed in ethanol (5 mL, 60% v/v) and ground. Following 60°C shock for 2 h, the homogenate was centrifuged at 120,00 r/min for 10 min at 4°C. The supernatant was removed and the total flavonoids were extracted. The total flavonoid content was measured using a NaNO2–AlCl3–NaOH method. Briefly, extracts (5 mL) were added in NaNO2 (5% w/v, 0.3 mL). After the mixture was agitated for 6 min, AlCl3 (10% w/v, 0.3 mL) was added and reacted for 6 min. NaOH (10% w/v, 4 mL) solution and 60% ethanol were then added to 10 mL of the mixture and shaken well for 10 min. Absorbance readings were taken at 510 nm using a spectrometer. Total flavonoid content was calculated based on a standard curve and expressed as mg of rutin (Zhu et al., 2022).

#### 2.2.2 Determination of iridoid terpenoids

Adjusted according to the method of Tian et al. (2018). A dry sample (0.1 g) passed through screen No. 4 was mixed in methanol (50 mL, 50% v/v), left for 30 min, and subjected to ultrasonic treatment (300 W, 40 kHz) for 30 min. The overall quality remained unchanged. After supernatant filtration, the sample was filtered by a 0.22 μm microporous filter membrane and used as the test solution. A total of 0.024, 0.0615, and 0.019 mg/ml mixed reference solutions of geniposidic acid, 8-epiloganic acid, and loganic acid, respectively, were prepared and filtered by a 0.22 μm filter membrane for reserve use. The standard curve was plotted to calculate the regression equation, with the concentration as the horizontal coordinate (X) and the peak area as the vertical coordinate (Y). The chromatographic conditions were as follows: column C18 (4.6 mm × 150 mm, 5 μm); mobile phase, acetonitrile (B) and 0.1% formic acid aqueous solution (A); gradient elution procedure (0–15 min, 5–9%B; 15–20 min, 9–19%B; 20–25 min, 19–22%B; 25–30 min, 22–25%B; 30–35 min, 25–30%B; 35–40 min, 30–5%B; 40–45 min, 5%B); volume flow rate, 0.8 mL/min; column temperature, 30°C; injection volume, 10 μL; and detection wavelength, 237 nm.

#### 2.2.3 Determination of polysaccharide

A dry sample (0.05 g) passed through screen No. 4 was homogenized in water (1 mL). The supernatant was extracted in a water bath at 100°C for 2 h, cooled and centrifuged at 10000 g for 10 min. Following this, 0.8 mL anhydrous ethanol was slowly added to 0.2 mL supernatant, mixed well, left to stand overnight at 4°C, and centrifuged at 10,000 g for 10 min. The supernatant was discarded, and 1 mL water was added to the precipitation to dissolve and precipitate. Phenol (5%w/v, 100 μL) and concentrated sulfuric acid (500 μL) were added into the 200 μL sample solution, heated in a water bath for 20 min, and then cooled under water. The absorbance was determined at 490 nm. The polysaccharide content was calculated according to the standard curve, and anhydrous glucose was used as the control substance (Ma et al., 2012).

### 2.3 Metabolomic analysis

In order to investigate the difference of metabolites between fresh oil cistanche and cistanche, the metabolic analysis of oil cistanche and cistanche squamata samples was performed. Sample Metabolite Extraction and Preparation: Tissues (100 mg) were individually grounded with liquid nitrogen and the homogenate was re- suspended with pre chilled 80% methanol and 0.1% formic acid by well vortex. The samples were incubated on ice for 5 min and then were centrifuged at 15,000 g, 4°C for 20 min. Some of supernatant was diluted to final concentration containing 53% methanol by LC-MS grade water. The samples were subsequently transferred to a fresh Eppendorf tube and then were centrifuged at Sample Metabolite Extraction and Preparation.

LC-MS/MS analyses were performed using an ExionLC™ AD system (SCIEX) coupled with aQTRAP^®^ 6500+ mass spectrometer (SCIEX) in Genedenovo (Guangzhou, China). Samples were injected onto a Xselect HSST3 (2.1×150 mm, 2.5 μm) using a 20-min linear gradient at a flow rate of 0.4 mL/min for the positive/negative polarity mode. The eluents were eluent A (0.1% Formic acid-water) andeluent B (0.1% Formic acid-acetonitrile). The solvent gradient was set as follows: 2% B, 2 min; 2–100% B, 15.0 min; 100% B, 17.0 min;100–2% B, 17.1 min;2% B, 20min. QTRAP^®^ 6500+ mass spectrometer was operated in positive polarity mode with Curtain Gas of 35 psi, Collision Gas of Medium, IonSpray Voltage of 5500V, Temperature of 550°C, Ion Source Gas of 1 :60, Ion Source Gas of 2 :60. QTRAP^®^ 6500+ mass spectrometer was operated in negative polarity mode with Curtain Gas of 35 psi, Collision Gas of Medium, Ion Spray Voltage of -4500V, Temperature of 550°C, Ion Source Gas of 1 :60, Ion Source Gas of 2 :60 (Want et al., 2012; Luo et al., 2015).

The detection of the experimental samples using MRM (Multiple Reaction Monitoring) were based on house database. The Q3 were used to the metabolite quantification. The Q1, Q3, RT (retention time), DP (declustering potential) and CE (collision energy) were used to the metabolite identification. The data files generated by HPLC-MS/MS were processed using the SCIEX OS Version 1.4 to integrate and correct the peak. The main parameters were set as follows: minimum peak height, 500; signal/noise ratio, 5; gaussian smooth width, 1. The area of each peak represents the relative content of the corresponding substance.

Finally, imultivariate statistical Analysis was adopted to conduct Principal Components Analysis on metabolite data of two samples. PCA), Partial Least Squares Discriminant Analysis (PLS-DA) and Orthoonal Partial Least Squares Discriminant analysis (OPLS-DA). Variable importance in the projection (VIP), Fold change value and P-value of OPLS-DA model were used to screen out the differential metabolites of Cistanche and Cistanche deserticola. The KEGG pathway of differential metabolites was analyzed. In this study, VIP≥1 and T-test P<0.05 in the OPLS-DA model were used as screening criteria for differential metabolites.

### 2.4 Transcriptomic analysis

Total RNA was extracted from 100 mg of fresh samples was extracted using the Trizol reagent kit (Invitrogen, Carlsbad, CA, USA). The RNA quality was assessed using an Agilent 2100 Bioanalyzer (Agilent Technologies, Palo Alto, CA, USA) and confirmed by agarose gel electrophoresis in the absence of RNase. After extraction, eukaryotic mRNA was enriched using Oligo(dT) beads, while prokaryotic mRNA was treated with the Ribo-ZeroTM Magnetic Kit (Epicentre, Madison, WI, USA) to remove rRNA. The enriched mRNA was then fragmented into short segments using fragmentation buffer and reverse-transcribed into cDNA with random primers. Second-strand cDNA was synthesized using DNA polymerase I, RNase H, dNTPs, and buffers. The cDNA fragments were then purified using a QiaQuick PCR Extraction Kit (Qiagen, Venlo, The Netherlands), followed by end repair, the addition of a single base, and ligation to Illumina sequencing adapters. The ligation products were then subjected to agarose gel electrophoresis, PCR amplification, and sequencing (Grabherr et al., 2011).

RNA differential expression analysis between the two groups was performed using DESeq2 (Wang et al., 2010). We employed |log2(fold-change)| > 1.5 and *P* < 0.05 as the criteria to identify differentially expressed genes between oil cistanche and Cistanche based on the differential expression level of each transcript. According to the significantly differentially expressed genes in each comparison group, these genes were compared to the GO (https://www.geneontology.org/) and KEGG (https://www.genome.jp/kegg/) databases to further determine DEG types. After removing unidentified and redundant DEGs by comparisons with the Swissport database (http://www.expasy.ch/sprot), the Uniprot database (https://www.uniprot.org/) was used to retrieve and categorize the biological and molecular functions of the identified and non-redundant DGEs.

### 2.5 Combined transcriptome and metabolome analysis

Based on the metabolite contents and gene expression values of oil cistanche and cistanche, we analyzed the DEGs and DAMs related to terpene synthesis pathways, phenylpropanoid glycosides, and carbohydrate-related pathways. In addition, to gain a deeper understanding of the interaction between the transcriptome and metabolome, the DEGs and DAMs were mapped to the KEGG pathway database to collect information on their shared pathways.

### 2.6 qRT-PCR Validation of DEGs

To validate the reliability of the transcriptome data, the relevant genes were selected for quantitative real-time-polymerase chain reaction (qRT-PCR) verification to determine their expression levels. *GAPDH* was used as the internal reference gene and primers were designed using the NCBI Primer-BLAST website (https://www.ncbi.nlm.nih.gov/tools/primer-blast/) (Li et al., 2021). The primer synthesis was performed by Shengon Biotech Co., Ltd (Shanghai, China). Fresh samples were grinded into powder in liquid nitrogen, and total RNA was extracted from 100 mg of this fresh powdered sample using the Plant RNA Kit R6827 produced by Omega. Reverse transcription to cDNA was performed using the FastKing RT Kit (KR116) from Tiangen Biotech (Beijing) Co., Ltd. qRT-PCR quantification was conducted using the SuperReal PreMix Plus (SYBR Green) (FP205) kit, also from Tiangen Biotech (Beijing) Co., Ltd. The qRT-PCR quantification was performed using an FTC-3000P Real-Time PCR System with the following reaction program: denaturation at 95°C for 10 min; 40 cycles of 95°C for 15 s, 55°C for 20 s, and 72°C for 30 s. Each sample was repeated three times, and the relative gene expression levels were calculated using the 2^-ΔΔ^Ct method (Livak and Schmittgen, 2001).

### 2.7 Statistical analysis

The mean and standard deviation represent the average of three measurements. A significant difference is considered at *P* < 0.05. Statistical analysis was performed using SPSS 22.0, while basic data processing was done using Excel software. GraphPad Prism software was utilized for creating bar charts.

## 3 Results

### 3.1 The determination of total flavonoid content in oil cistanche and cistanche

**Figure 2:**
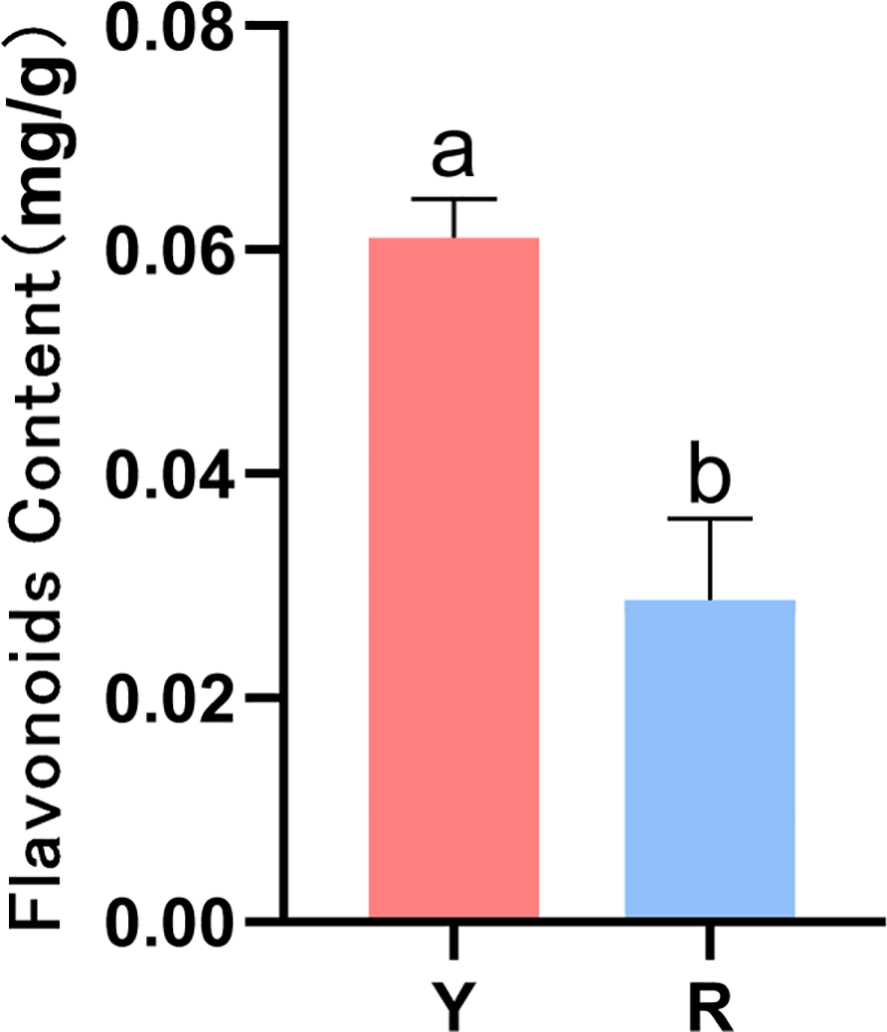
Contents of flavonoids in oil cistanche (Y) and Cistanche (R)(mean ± SD, n = 3). the “a, b” is considered significant at *p* < 0.05 between Y and R.

The content of total flavonoids of fresh oil cistanche and cistanche samples was determined, and the content of total flavonoids in oil cistanche was 2.13 times higher than that in cistanche (**Figure 2**).

### 3.2 Metabolomic analysis

In order to elucidate the specific metabolic mechanisms behind the color differences between fresh oil cistanche and cistanche, this study conducted an extensive targeted metabolic analysis on the squamate stem samples of both species. A total of 959 metabolites were found in 20 classes, including 186 amino acids and their derivatives, 72 nucleotides and their derivatives, 110 sugars and their derivatives, 8 alcohols and polyols, 91 organic acids and their derivatives, 20 vitamins, 112 and lipids. Secondary metabolites include 38 terpenoids, 33 alkaloids and their derivatives, 29 phenylpropanoid and polyketide compounds, 6 benzenes and their substituted derivatives, 32 phenols and their derivatives, 34 phenolic acids, 68 flavonoids, and 15 phytohormones. Based on the PCA and OPLS-DA results, PC1 and PC2 were determined to contribute 30.2% and 22.1%, respectively, and the two samples were well distinguished.

**Figure 3:**
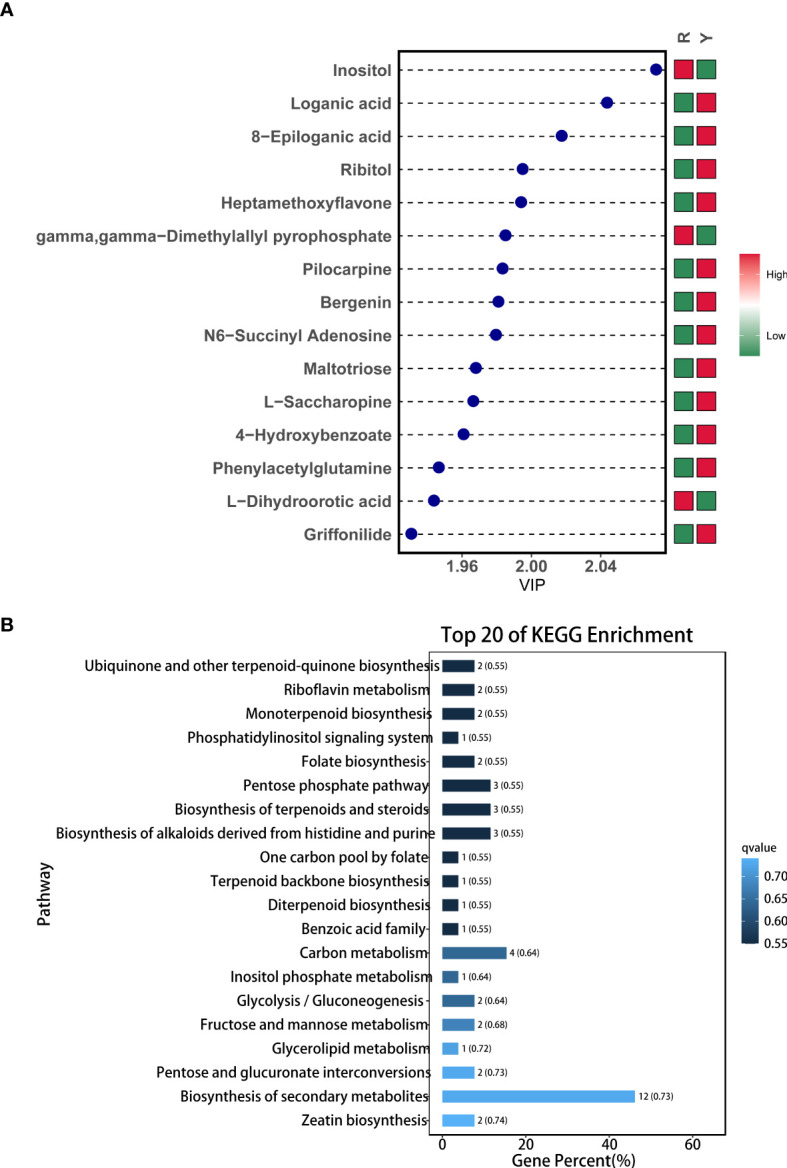
Metabolite profiles between Y and R. **(A)** Variable Importance in Projection chart of DAMs; **(B)** KEGG enrichment of DAMs.

The data processing and mapping of differential metabolites showed that there were a total of 50 DAMs, of which 34 were UR and 16 were DR (**Table 1**). It included 4 flavonoids, 11 carbohydrates and its derivatives, 5 terpenoids, 7 amino acid and its derivatives, 3 nucleotide and its derivates, 6 organic acid and its derivatives, 7 organoheterocyclic compounds, 3 phytohormones, 2 alcohols and polyols, and 1 phenols and its derivatives, alkaloids and derivatives, and lipids. Among them, the top 15 metabolites with the highest VIP values are shown in **Figure 3A**. KEGG enrichment of differential metabolites showed that the first 20 pathways mainly included “Ubiquinone and other terpenoid-quinone biosynthesis”, “monoterpenoid biosynthesis” and “Biosynthesis of terpenoids and steroids” (**Figure 3B**).

**Table 1:** Classification of DAMs and their differential accumulation levels in R vs. Y (mean ± SD, n=3).

No.	Class	Compounds Name	Formula	log2FC
1	Flavonoids	Heptamethoxyflavone	C_22_H_24_O_9_	1.596530003
2		Apigenin 5-O-glucoside	C_21_H_20_O_10_	1.447270251
3		Chrysoeriol 7-O-hexoside	C_22_H_22_O_11_	1.370956862
4		di-C,C-hexosyl-methylluteolin	C_28_H_32_O_16_	0.731838968
5	Carbohydrates And Its Derivatives	Prim-O-glucosylcimifugin	C_22_H_28_O_11_	2.333384812
6		Lobetyolin	C_20_H_28_O_8_	1.617890962
7		Maltotriose	C_18_H_32_O_16_	1.187420217
8		phosphoribosyl pyrophosphate	C_5_H_13_O_14_P_3_	1.072165515
9		Ribitol	C_5_H_12_O_5_	0.768077062
10		D-Xylulose	C_5_H_10_O_5_	-0.484836655
11		L-Sorbose	C_6_H_12_O_6_	-0.692733624
12		D-Galactose	C_6_H_12_O_6_	-0.781704466
13		Fructose bisphosphate	C_6_H_14_O_12_P_2_	-1.420545477
14		D-Fructose-1,6-biphosphate	C_6_H_14_O_12_P_2_	-1.443818607
15		D-3-Phosphoglyceric acid	C_3_H_7_O_7_P	-1.449220241
16	Terpenoids	Loganic acid	C_16_H_24_O_10_	2.448518374
17		8-Epiloganic acid	C_16_H_24_O_10_	1.919374294
18		Geniposidic acid	C_16_H_22_O_10_	1.383149201
19		Diosgenin	C_27_H_42_O_3_	-0.103390846
20		Ajugol	C_15_H_24_O_9_	-0.742785267
21	Amino Acid And Its Derivatives	N-(Phenylacetyl)-L-phenylalanine	C_17_H_17_NO_3_	1.990506548
22		Phenylacetylglutamine	C_13_H_16_N_2_O_4_	1.737855689
23		Dl-2-Aminooctanoic Acid	C_8_H_17_NO_2_	1.016689663
24		L-Saccharopine	C_11_H_20_N_2_O_6_	0.787699404
25		4-Acetamidobutyric Acid	C_6_H_11_NO_3_	0.613913384
26		L-Dihydroorotic acid	C_5_H_6_N_2_O_4_	-0.585123705
27		Nicotianamine	C_12_H_21_N_3_O_6_	-1.365123664
28	Organic Acid And Its Derivatives	3-Hydroxybutyrate	C_4_H_8_O_3_	2.770608595
29		Homogentisic Acid	C_8_H_8_O_4_	2.006082946
30		Bergenin	C_14_H_16_O_9_	1.764766976
31		4-Hydroxybenzoate	C_7_H_6_O_3_	1.559592207
32		2-(Formylamino)benzoic acid	C_8_H_7_NO_3_	1.210922373
33	Alcohols and polyols	Patchouli alcohol	C_15_H_26_O	-0.440339709
34		Inositol	C_6_H_12_O_6_	-1.695632363
35	Phenols And Its Derivatives	Androsin	C_15_H_20_O_8_	2.111889472
36	Nucleotide And Its Derivates	N6-Succinyl Adenosine	C_14_H_17_N_5_O_8_	1.288666118
37		Uridine 5’-Diphospho-N-Acetylgalactosamine	C_17_H_27_N_3_O_17_P_2_	-0.575550096
38		UDP-D-xylose	C_14_H_22_N_2_O_16_P_2_	-0.644147008
39	Organoheterocyclic compounds	Griffonilide	C_8_H_8_O_4_	2.841063021
40		1-Methylnicotinamide	C_7_H_9_N_2_O+	2.020807033
41		10-Formyl-THF	C_20_H_23_N_7_O_7_	1.358362619
42		4-Hydroxyquinazoline	C_8_H_6_N_2_O	1.019823465
43		3-Indoleacrylic acid	C_11_H_9_NO_2_	0.815885719
44		Purine	C_5_H_4_N_4_	0.638248526
45		Rhodomyrtone	C_26_H_34_O_6_	-0.108492437
46	Lipids	gamma,gamma-Dimethylallyl pyrophosphate	C_5_H_12_O_7_P_2_	-1.942820847
47	Phytohormones	Gibberellin A3	C_19_H_22_O_6_	3.179982394
48		trans-zeatin N-glucoside	C_16_H_23_N_5_O_6_	2.086869061
49		Salicylic acid O-glucoside	C_13_H_16_O_8_	1.60165752
50	Alkaloids and derivatives	Pilocarpine	C_11_H_16_N_2_O_2_	2.36136598

### 3.3 Transcriptomic analysis

#### 3.3.1 Sample quality control analysis

In order to investigate the transcriptome differences between oil cistanche and cistanche, second-generation transcriptome sequencing was performed on fresh oil cistanche and cistanche squamous stem samples. In order to ensure data quality, we filter the original data before information analysis to reduce the interference of invalid data. After filtering the low-quality data from the original data, the base composition and mass distribution were more balanced, indicating that the filtered transcripts were of higher quality and could ensure the accuracy of subsequent analysis. Two standardized cDNA libraries were constructed from the RNA of Y and R. After filtering and the identification of the cDNA library, 41.25 and 37.65 million high-quality reads were collected. the Q20 reads of the Y and R were 98.70% and 98.69%, respectively (0.3% error probability). The GC content of the reads was approximately 44.97% and 44.91%, respectively, and the measured gene expression levels were reliable.

#### 3.3.2 Differentially expressed genes analysis

**Figure 4:**
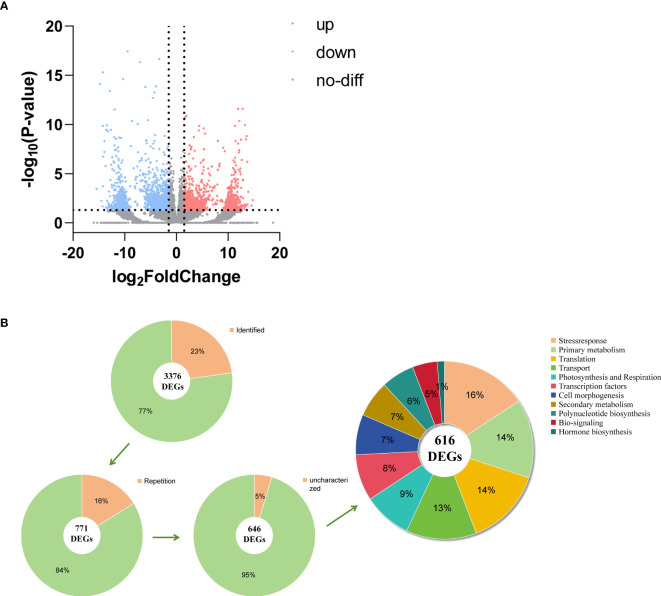
DEGs analysis between oil cistanche and cistanche. **(A)** volcano plot comparing oil cistanche and cistanche; **(B)** gene classification statistical chart.

A total of 105,720 sequences of transcripts were compared and functionally annotated in the four major databases NR, KEGG, KOG, and SwissProt, and 39,859 transcripts were annotated. Among them, 39,115 full-length NR transcripts were annotated in the protein database, 38,151 in KEGG, 20,583 in KOG, and 20,757 in SwissProt. By comparing the transcription between oil cistanche and cistanche, we obtained 3,376 DGEs from 39,859 unigenes, including 1,524 UR and 1,852 DR genes (**Figure 4A**). Based on further classification of DEGs, 2,606 genes from 3,376 DEGs were not identified in the SwissProt database. We removed 114 duplicate genes and 42 of the 657 identified DEGs without biological characteristics. The remaining 616 have known functions and can be classified into 11 categories: 54 Photosynthesis and respiration genes, 88 Primary metabolism genes, 41 Secondary metabolism genes, 8 Hormone biosynthesis genes, 45 Cell morphogenesis genes, 28 Bio-signaling genes, 37 Polynucleotide biosynthesis genes, 52 Transcription factors genes, 87 Translation genes, 79 Transport genes, 97 Stress response genes (**Figure 4B**).

#### 3.3.3 Functional annotation and enrichment of DEGs

**Figure 5:**
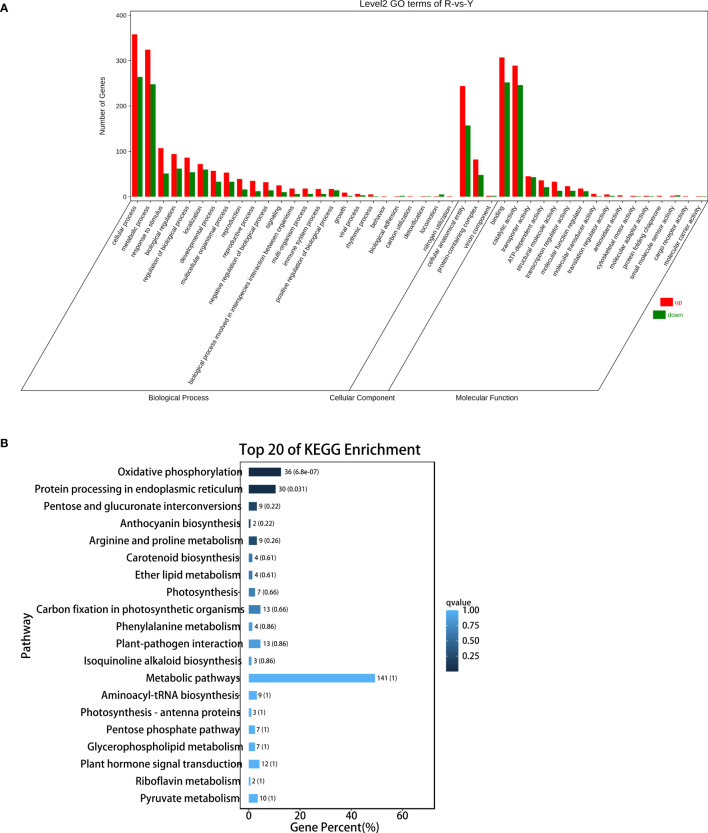
DEGs analysis between oil cistanche and cistanche. **(A)** gene ontology (GO) enrichment of DEGs; **(B)** KEGG enrichment of DEGs.

The function of the 3376 DEGs was annotated against the Gene Ontology (GO) and KEGG databases. For the GO database, 53 terms were classified into biological process, cellular component, and molecular function (**Figure 5A**). The top 20 KEGG pathways were analyzed, and the top 10 pathways including: oxidative phosphorylation; Oxidative phosphorylation; Protein processing in endoplasmic reticulumPentose and glucuronate interconversions; Anthocyanin biosynthesis; Arginine and proline metabolism; Carotenoid biosynthesis; Ether lipid metabolism; Photosynthesis; Carbon fixation in photosynthetic organisms; Phenylalanine metabolism (**Figure 5B**).

### 3.4 Combined analysis of DEGs and DAMs

**Figure 6:**
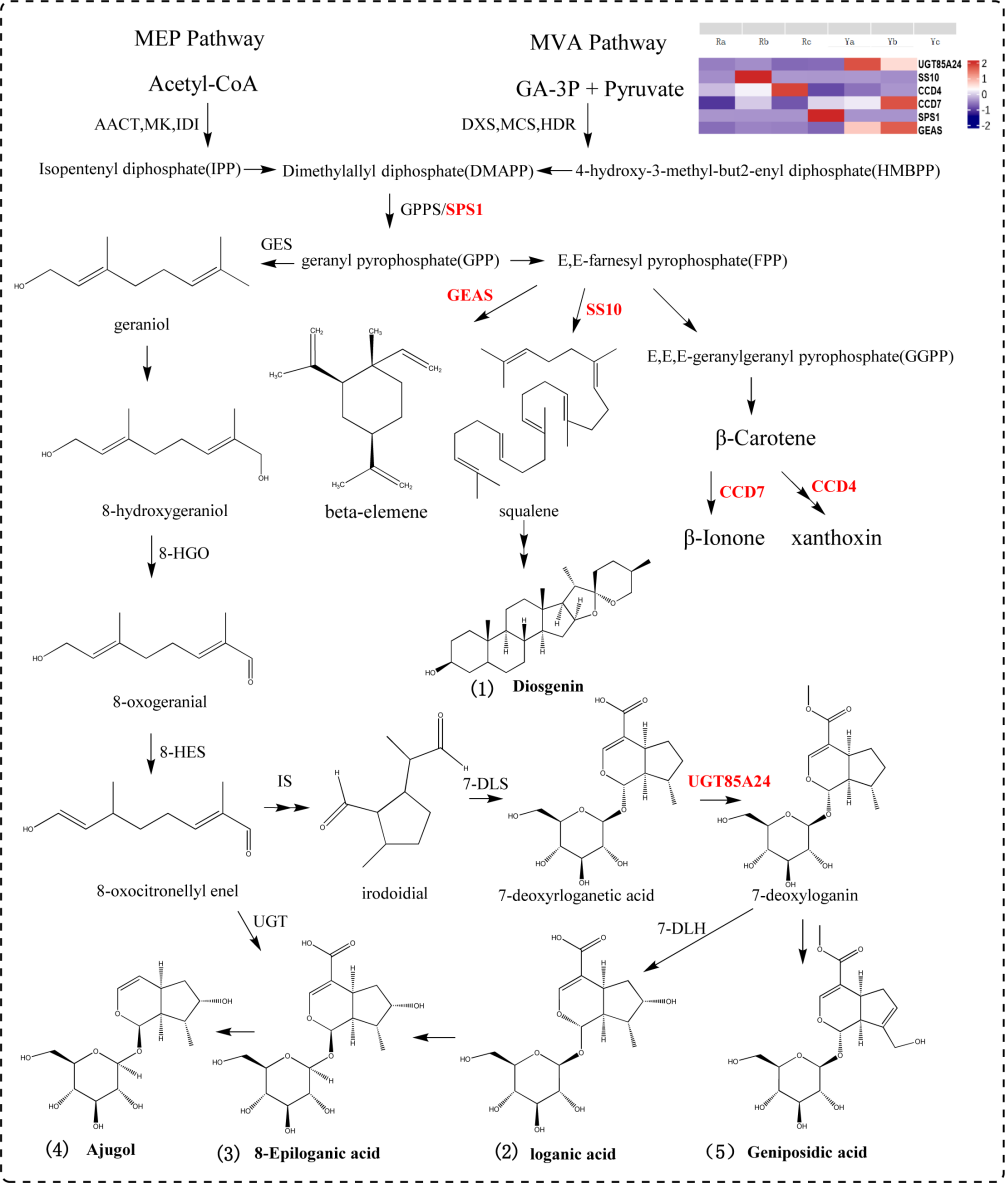
Terpenoids synthetic pathway diagram.

A precursor of terpenoid compounds, IPP, is synthesized from the mevalonate and methylerythritol phosphate pathways. IPP and diallyl pyrophosphate are condensed under the catalysis of geranyl diphosphate (GPP) by geranyl diphosphate synthase (Singh et al., 2021; Miettinen et al., 2014; Rodríguez-López et al., 2022; Palmer et al., 2022). GPP is the dividing point for terpenes to form different compounds such as monoterpenes, diterpenes, triterpenes and alkaloids (Zhang et al., 2022; Yoshidome et al., 2023; Burse et al., 2007). In this study, seven DEGs (*SPS1, UGT85A24, UGT87A2, UGT9, GEAS, SS10, CCD4*,and *CCD7*) and five DAMs (loganic acid, 8-epigylanic acid, geniposidic acid, ajugol, and diosgenin) were selected to participate in the terpenoid synthesis pathway. *SPS1* catalyzes the conversion of dimethylallyl diphosphate (DMAPP) to GPP. *SS10* is a key gene in the synthesis of squalene, which controls the formation of triterpenoids and can be used to synthesize diosgenin under subsequent catalysis. *GEAS* mediates the conversion of (2E,6E) -farnesyl diphosphate (FPP) to germacrene A and beta-elemene to synthesize sesquiterpene compounds. *CCD4* is involved in the cleavage of carotenoids, and *CCD7* breaks down β-carotene to produce β-ionone. In this pathway, 7-deoxyadenosine is converted into 7-deoxyloganin by the action of *UGT85A24*. 7-deoxyloganin is further transformed into loganic acid, 8-epiloganic acid and ajugol by 7-deoxyloganic acid hydroxylase (*DL7H*). *UGT85A24* acts on genipin and 7-deoxyloganetin, and participates in geniposide biosynthesis (**Figure 6**).

**Figure 7:**
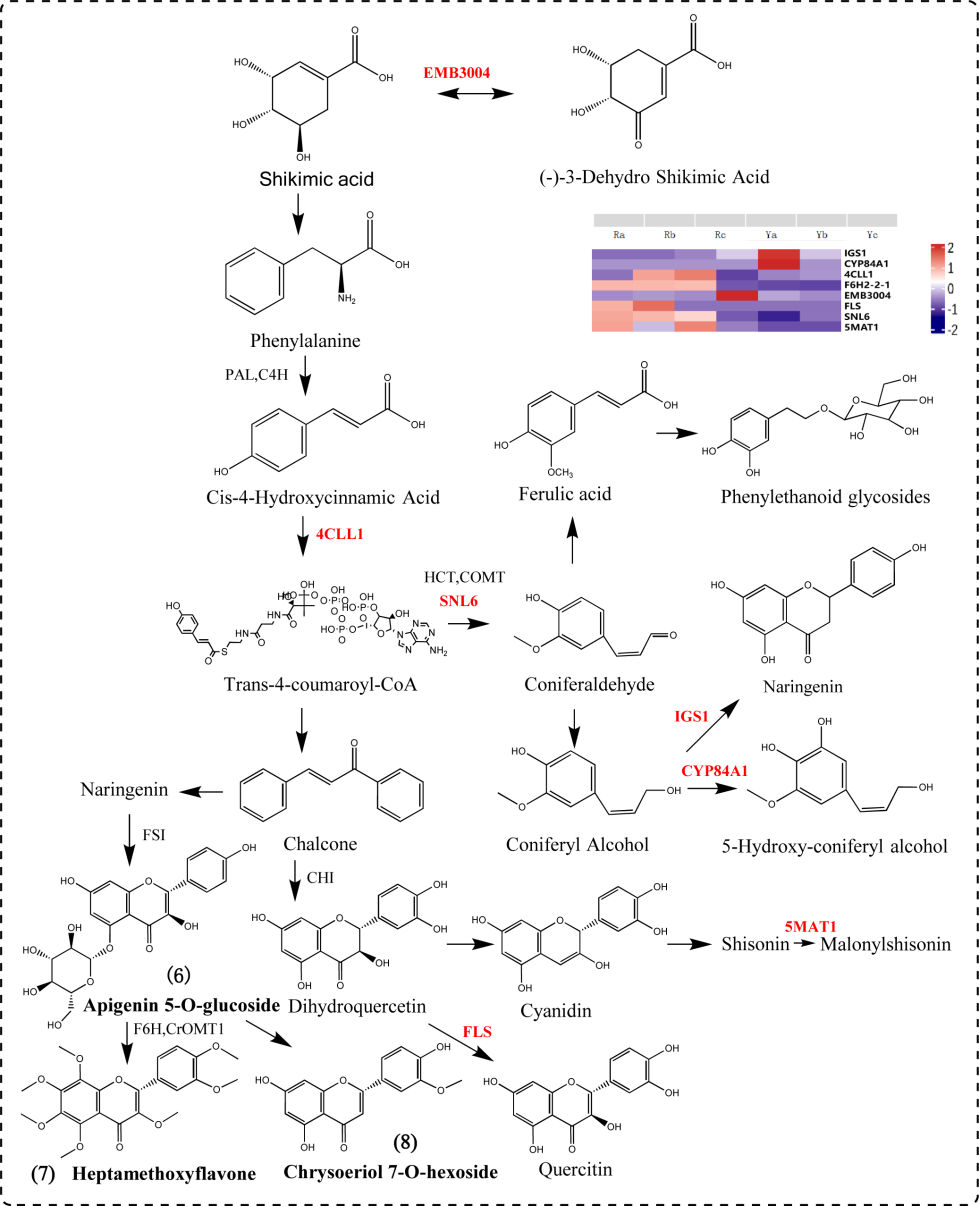
Phenylpropanoid biosynthesis pathway diagram.

We screened out seven DEGs (*4CLL1, F6H2-2-1, FLS, CYP84A1, IGS1, SNL6*, and *5MAT1*) involved in the phenylpropanoid biosynthesis pathway. Phenylalanine is catalyzed by *PAL* and *4CLL1* to form p-coumaryl CoA, the upstream metabolite of phenylpropyl synthesis. *CYP84A1* is a member of the CYP84 (also known as ferulate acid 5-hydroxylase (F5H)) subfamily of cytochrome P450-dependent monooxygenase and is an indispensable enzyme in anthocyanin biosynthesis and accumulation-related gene expression (Tetreault et al., 2020; Nair et al., 2000; Anderson et al., 2015; Maruta et al., 2014). *5MAT1* participates in the anthocyanin biosynthesis pathway, catalyzing the conversion of shisonin to malonylshisonin. *FLS* catalyzes the formation of flavonols from dihydroflavonols. Chalcones produce naringin, apigenin is produced under the catalysis of *FSI*, apigenin derivatives are produced, and then chrysoserin is synthesized. Apigenin and lysionotin produce heptamethoxyflavone under the action of *F6H, CrOMT1* and other genes (Peng et al., 2024). *IGS1* can catalyze the formation of naringetol from coniferyl alcohol (**Figure 7**).

**Figure 8:**
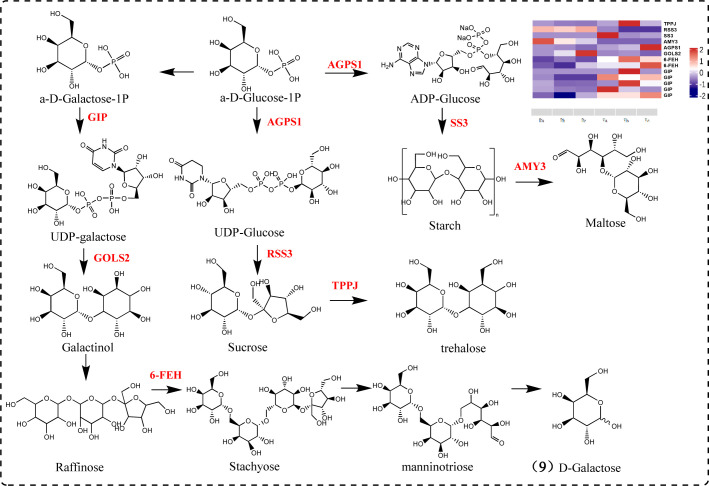
Galactose metabolism and starch and sucrose metabolism pathway diagram.

Polysaccharides are composed of various monosaccharides, including glucose, mannose, galactose, galacturonic acid, arabinose, and rhamnose. Sucrose is catalyzed by sucrose synthase to produce UDP-glucose, while UDP-galactose is generated through the galactose metabolic pathway. Both UDP-glucose and UDP-galactose are components of polysaccharides (Xiao and Su, 2023). Our results indicate significant differences between oil cistanche and cistanche in the expression of genes related to galactose metabolic pathways and starch and sucrose metabolic pathways. Among these, *TPPJ, AGPS1, SS3*, and *6-FEH* are UR, while *RSS3, AMY3*, and *GOLS2* are DR. *RSS3*, a sucrose-splitting enzyme, provides UDP-glucose and fructose for various metabolic pathways (Lee et al., 2007). *AGPS1* catalyzes the synthesis of ADP-glucose, which is converted into starch sugars and subsequently participates in the synthesis of starch under the promotion of *SS3* (Piro et al., 2023). *AMY3* participates in starch catabolic metabolism. *TPPJ* catalyzes the production of trehalose. *GIP* catalyzes the synthesis of UDP-galactose. *GOLS2* participates in the synthesis of raffinose, a member of the raffinose family of oligosaccharides (RFO) (Sprenger and Keller, 2000). *GOLS2* converts UDP-galactose into galactinol, which is then converted into raffinose by RFS. *6-FEH* generates melibiose and mannotriose, promoting the production of D-galactose. In addition, the *6-FEH* gene can hydrolyze fructan-type β-(2->6)-linked fructans into fructose (Van den Ende et al., 2003). These genes play a key role in the galactose metabolism and starch and sucrose metabolism pathways, and influence polysaccharide formation under the action of subsequent glycosyltransferases (**Figure 8**).

### 3.5 Validation by qRT–PCR

**Figure 9:**
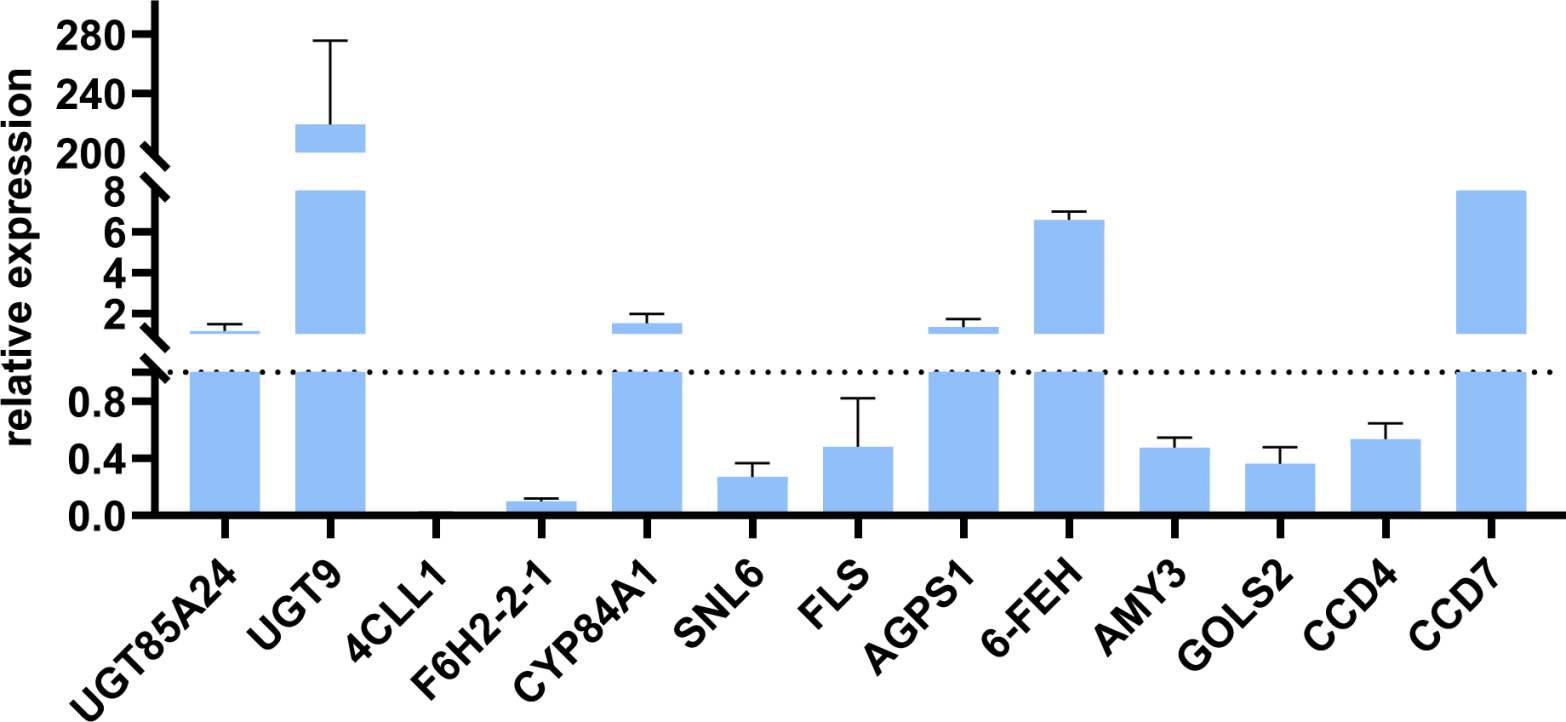
The Relative Expression of genes involved in Terpenoids synthetic pathway, phenylpropanoid biosynthesis pathway, starch and sucrose metabolism pathway, and Galactose metabolism pathway in oil cistanche and cistanche, as determined by qRT-PCR (mean ± SD, n=3).

The key genes for the terpenoids synthetic pathway, phenylpropanoid biosynthesis pathway, starch and sucrose metabolism pathway, and galactose metabolism pathway (*UGT85A24, UGT9, 4CLL1, F6H2-2-1, CYP84A1, SNL6, FLS, AGPS1, 6-FEH, AMY3, GOLS2, CCD4* and *CCD7*) were selected for qRT–PCR validation (n = 3). The results show that the changes of 13 key genes in oil cistanche and cistanche are generally consistent with the gene expression trends obtained from the transcriptome sequencing, indicating a high reliability of the sequencing data in this study. Among them, genes *UGT85A24*, *UGT9* and *CCD7*, which are involved in the synthesis of terpenoid components, were upregulated by 1.15-, 218.98-, and 9.56-fold, respectively. involved in the phenylpropanoid biosynthesis, was upregulated by 1.53-fold, and *4CLL1, F6H2-2-1, SNL6*, and *FLS* were downregulated by 0.02-, 0.10-, 0.27-, and 0.48-fold, respectively. Genes *AGPS1* and *6-FEH*, which are involved in the starch and sucrose metabolism pathway and galactose metabolism pathway, exhibited a 1.33 and 6.59-fold upregulation, respectively, while the genes *AMY3* and *GOLS2* exhibited a 0.48 and 0.36-fold down-regulation, respectively (**Figure 9**).

### 3.6 Determination of main active components of dried oil cistanche and cistanche

**Figure 10:**
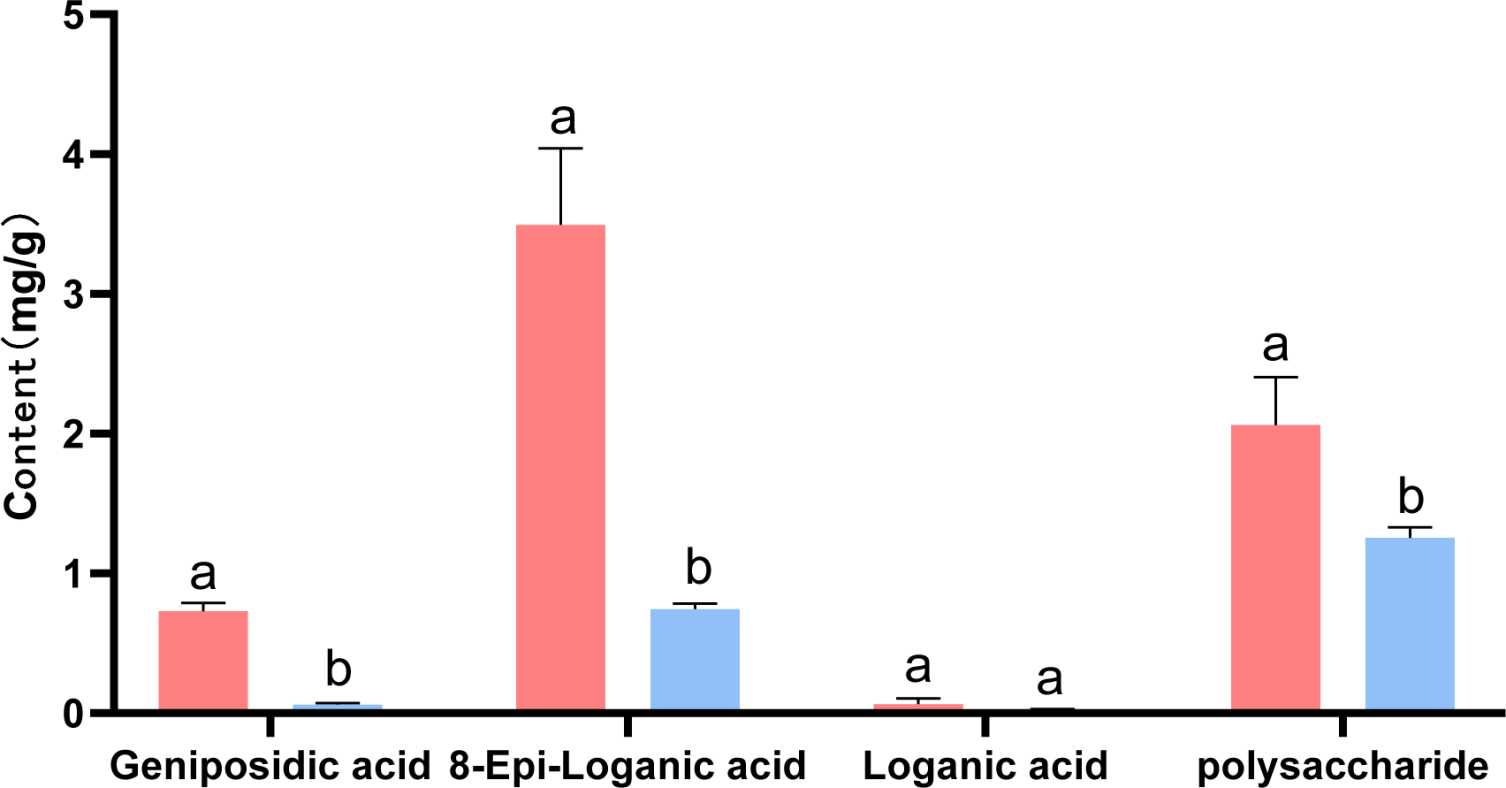
The content of major constituents (iridoids, and polysaccharides). The different lowercase letters (a, b and c) represent samples from three biological replicates.

Based on the results of transcriptome and metabolome analysis, we found that the synthesis pathways of terpenoids and polysaccharides in oil cistanche were significantly different from those in cistanche. Therefore, high-performance liquid chromatography was used for the determination of iridoid compounds in dried oil cistanche and cistanche samples and for the determination of polysaccharide content in both.The results showed that the contents of 8-epiloganic acid and geniposidic acid in oil cistanche were significantly higher than those in cistanche, being 4.70 and 12.01 times higher, respectively. The polysaccharides content of oil cistanche was also significantly higher than that of cistanche, being 1.64 times higher (**Figure 10**).

## 4 Discussion

The differences in apparent color among plants are manifestations of their physiological and biochemical variations (Piatkowski et al., 2020; Li et al., 2024). Typically, the variation in plant color is associated with flavonoid components, which are one of the most significant pigments in plants. In this study, the flavonoid content in fresh oil cistanche and cistanche was measured. The results revealed that the flavonoid content in purple oil cistanche was significantly higher than that in yellow cistanche. The flavonoid content positively contributes to pigmentation (Zhuang et al., 2019). This phenomenon is also observed in many similar plants, such as *Clematis tangutica* (Guo et al., 2023), *Camellia sinensis* (Liu et al., 2023), Purple Chinese Cabbage (Park et al., 2021), and *Salvia miltiorrhiza* (Yang et al., 2022). Additionally, flavonoid compounds play a crucial role in plant resistance to abiotic stress. An increase in flavonoid content enhances antioxidant properties, thereby significantly strengthening the plant’s resistance to salt, drought, and cold stress (Liu et al., 2022; Isshiki et al., 2014; Nakabayashi et al., 2014). We speculate that oil cistanche is more adapted to the arid and saline-alkali soils of northwest China.

In addition to flavonoids, plant color is also related to changes in the content of isoprenoid compounds, including iridoids, carotenoids, and quinones. Anthocyanins, belonging to the flavonoid class of compounds, are the primary pigments responsible for the red, purple, and blue hues in plants (Muñoz-Gómez et al., 2021; Alappat and Alappat, 2020). In this study, we employed metabolomics to analyze the metabolites contributing to the color differences between fresh samples of oil cistanche and cistanche. There are four flavonoid differential metabolites in oil cistanche and cistanche, namely, heptamethoxyflavone, apigenin 5-O-glucoside, chrysoeriol 7-O-hexoside, and di-C,C-hexosyl-methylluteolin. Among them, apigenin 5-O-glucoside has a higher content in blue-purple plant species (Mizuno et al., 2024, Mizuno et al., 2021). Moreover, di-C,C-hexosyl-methylluteolin belong to the C-glycosyl-anthocyanins, which are much more stable than C-O-linked anthocyanins and are widely used in coloring. Flavone O-glucoside, apiin, and some flavanone O-glycosides and reducing substances produce red to purple pigments (Bjorøy et al., 2009). This suggests that the accumulation of heptamethoxyflavone, apigenin 5-O-glucoside, chrysoeriol 7-O-hexoside, and di-C,C-hexosyl-methylluteolin are the primary metabolic reasons for the purple color of the squamate stems in oil cistanche. Terpenoids, including carotenoids and iridoids. Among them, geniposidic acid, an iridoid component, is a natural blue pigment. Geniposidic acid can react to form purple-red polymers and, when reacted with methylamine, produces blue-black and purple-red pigments. Carotenoids, also natural pigments, can supplement flavonoid/anthocyanin levels when they decline (Zhou et al., 2022). Differential metabolites, including loganic acid, 8-epiloganic acid, and geniposidic acid, are present at higher levels in oil cistanche compared to cistanche, which is also one of the reasons affecting the purple coloration of oil cistanche. The regulation of pigment biosynthesis is complex and influenced by species, environmental conditions, and their interactions. It is therefore speculated that the purple color of oil cistanche is conferred by the combined interaction of terpenoids and anthocyanins, which aligns with the findings by Zhou et al (2022).

In plants, the biosynthesis of anthocyanins is located downstream of the phenylpropanoid synthesis pathway. In this study, genes related to the phenylpropanoid synthesis pathway were identified. Among them, *4CLL1* is a key rate-limiting enzyme in the plant phenylpropanoid synthesis pathway, catalyzing the formation of 4-coumaroyl-CoA. Three genes are involved in the biosynthesis of anthocyanins, among which the upregulated gene *CYP84A1* participates in the biosynthesis and accumulation of anthocyanins (Maruta et al., 2014). The downregulated gene *5MAT1* catalyzes the conversion of cyanidin to malonyl-cyanidin, and *FLS* can catalyze the formation of flavonoids from dihydroflavonol. In addition, research in *Arabidopsis thaliana* has shown that anthocyanin production is stimulated by a specific sucrose signaling pathway (Wang et al., 2019) This study found that oil cistanche and cistanche have more differential genes in the sucrose and starch metabolic pathways, which may affect the anthocyanin production in oil cistanche. The *UGT85A24*, which is involved in the biosynthetic pathway of terpenoids, can promote the synthesis and accumulation of geniposidic acid (Adamenko et al., 2018; Nagatoshi et al., 2011). In this study, *UGT85A24* was upregulated in oil cistanche, acting on 7-deoxyadenosine to form 7-deoxyloganin, which then undergoes catalysis by *DL7H* to produce loganic acid, 8-epiloganic acid, and geniposidic acid. Consequently, the accumulation of the differential metabolites loganic acid, 8-epiloganic acid, and geniposidic acid was relatively high, which is correlated with the upregulation of *UGT85A24*. In the carotenoid synthesis pathway, the differential expression of *CCD4* and *CCD7* generates beta-carotene, which is an important metabolite for plants to produce the color purple (Rubio et al., 2008). Therefore, the fresh oil cistanche has a deep purple color, which is related to the content of flavonoids and terpenoid differential metabolites, as well as the differential expression of *UGT85A24, CCD4, CCD7, CYP84A1, 5MAT1* and *FLS*.

**Figure 11:**
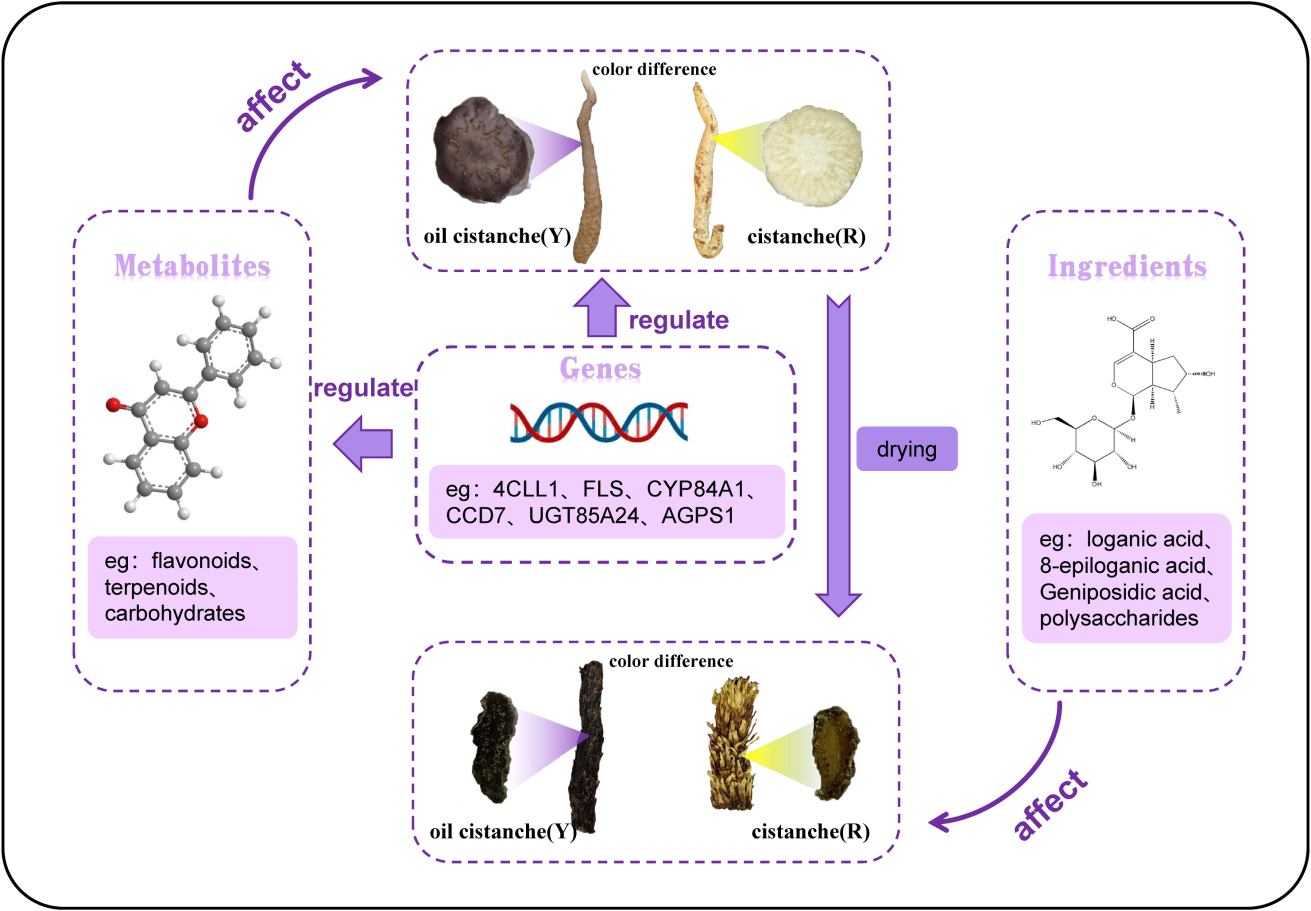
Schematic diagram of the formation mechanism of color difference between Y and R.

Fresh oil cistanche and cistanche showed a great difference in color, and this difference still existed in the dried samples, with the dried oil cistanche being darker in color compared to cistanche, and more popular in the market. On this basis, we also analysed the causes of the color difference between the dried oil cistanche and cistanche samples. Iridoids are reported to be active and prone to oxidation, hydrolysis, and transformation, and iridoid decomposition can lead to the deepening and blackening of medicinal materials. The HPLC results showed that the content of loganic acid in dried oil cistanche samples decreased, which is different from the high expression of the metabolic group. This may be caused by the transformation and decomposition of loganic acid in the oil cistanche samples during drying, which may also explain the relative blackness of oil cistanche after drying. The content of iridoids is significantly correlated with plant color, namely, the higher the content of iridoids, the darker the plant color (Li et al., 2018). Early studies suggest that the polysaccharide content can affect the color of traditional herbs (Li and Xu, 1990; Lai et al., 2019; Zhao et al., 2017), with polysaccharides identified as the material basis for the black appearance of cistanche deserticola, The contents of iridoids and polysaccharides in the dried oil cistanche were significantly higher than those in cistanche. The blacker color of oil cistanche may be attributed to the difference in iridoid and polysaccharide contents (**Figure 11**).

The change of cistanche from fresh to dry is a dynamic process. Early studies have shown that the dark appearance of cistanche squamata stems after processing is mainly related to the Maillard reaction, and the further deepening of the color of steamed cistanche after drying is caused by the decrease of pigment concentration. During the whole process of drying, the cell structure, composition, color, metabolic process and gene regulation of cistanche are all changing (Ai et al., 2022, Ai et al., 2021). In the future, We will study the drying kinetics characteristics of cistanche herb, establish an appropriate drying kinetics model, further evaluate different drying methods and the dynamic changes of effective components and colors during drying, and increase the research on cell structure, material metabolism and gene regulation of oil cistanche in the whole process from fresh to drying, so as to explain the changes of oil cistanche in the drying process.

At present, the few studies on oil cistanche are limited to determining the content of active ingredients after drying, while other important aspects are unexplored. We suggest that future research on oil cistanche should focus on using bionic technology to objectively compare the differences in appearance and traits between oil cistanche and cistanche. In particular, the application of widely targeted metabolomics can facilitate the analysis of metabolites in oil cistanche after drying, establishing animal experimental models for drug administration and index observations. Furthermore, scholars should explore the clinical efficacy of oil cistanche and conduct research on the growth, development, and stress resistance of oil cistanche plants, allowing for a comprehensive evaluation of oil cistanche.

## 5 Conclusion

In summary, the dark and purple color of fresh oil cistanche is related to the high content of total flavonoids, which is the result of the interaction between the differences in iridoid and flavonoid metabolites and differential expression of related synthesis regulatory genes. Dried samples of oil cistanche were darker in color than those of cistanche, due to the higher content of iridoids and polysaccharides in the former.

## Data Availability

The datasets presented in this study can be found in online repositories. The names of the repository/repositories and accession number(s) can be found in the article/**Supplementary Material**.
