# Supplementary material for: Transcriptomic and metabolomic analyses reveal the mechanism of color difference between two kinds of Cistanche deserticola before and after drying
Source: Front Plant Sci. 2025 Jan 23;15:1506523. doi: 10.3389/fpls.2024.1506523 (PMC11798998; doi:10.3389/fpls.2024.1506523)
Supplement: Supplementary file 1 [file Supplementaryfile1.docx]

**Figure supplementary legends**


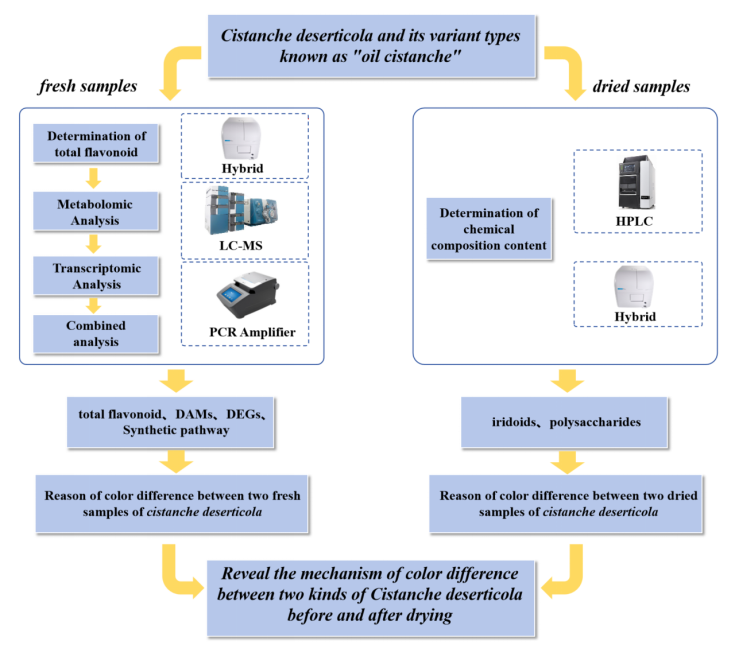


**Fig. S1** Graphical Abstracts.


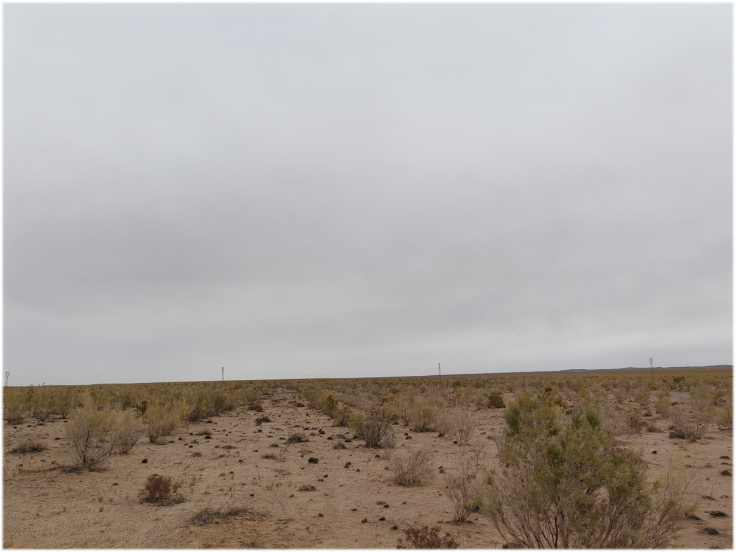


**Fig. S2** *Cistanche deserticola* plants grown in the planting site.


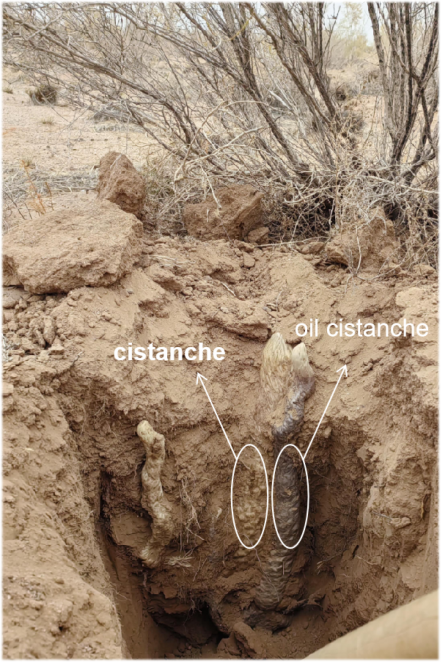


**Fig. S3** Comparison of oil Cistanche and cistanche deserticola in origin.


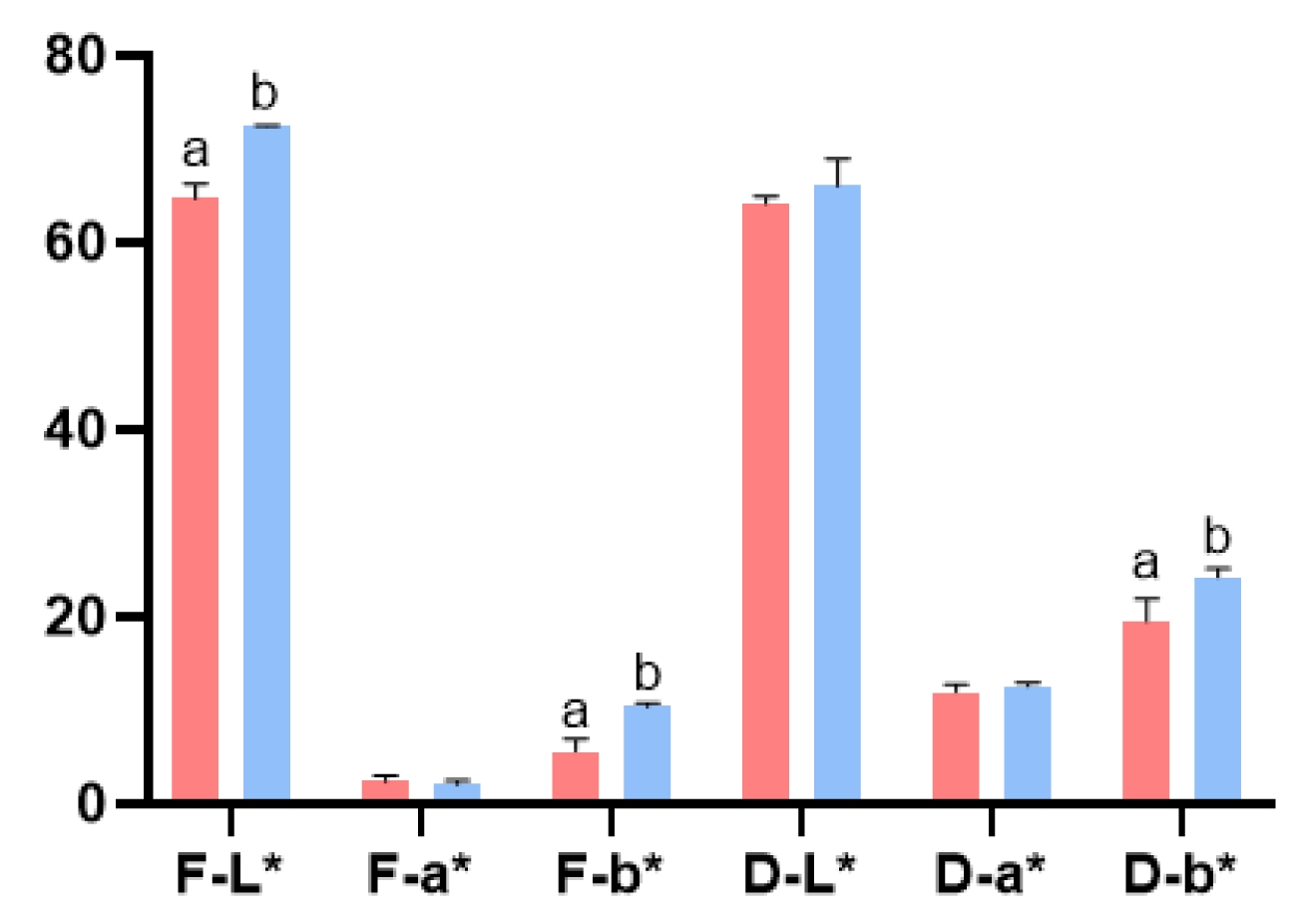


**Fig. S4** ANOVA for the parameter of bulb color. F for fresh samples, D for dried samples.（Different letters indicate significant differences, *P*<0.05）


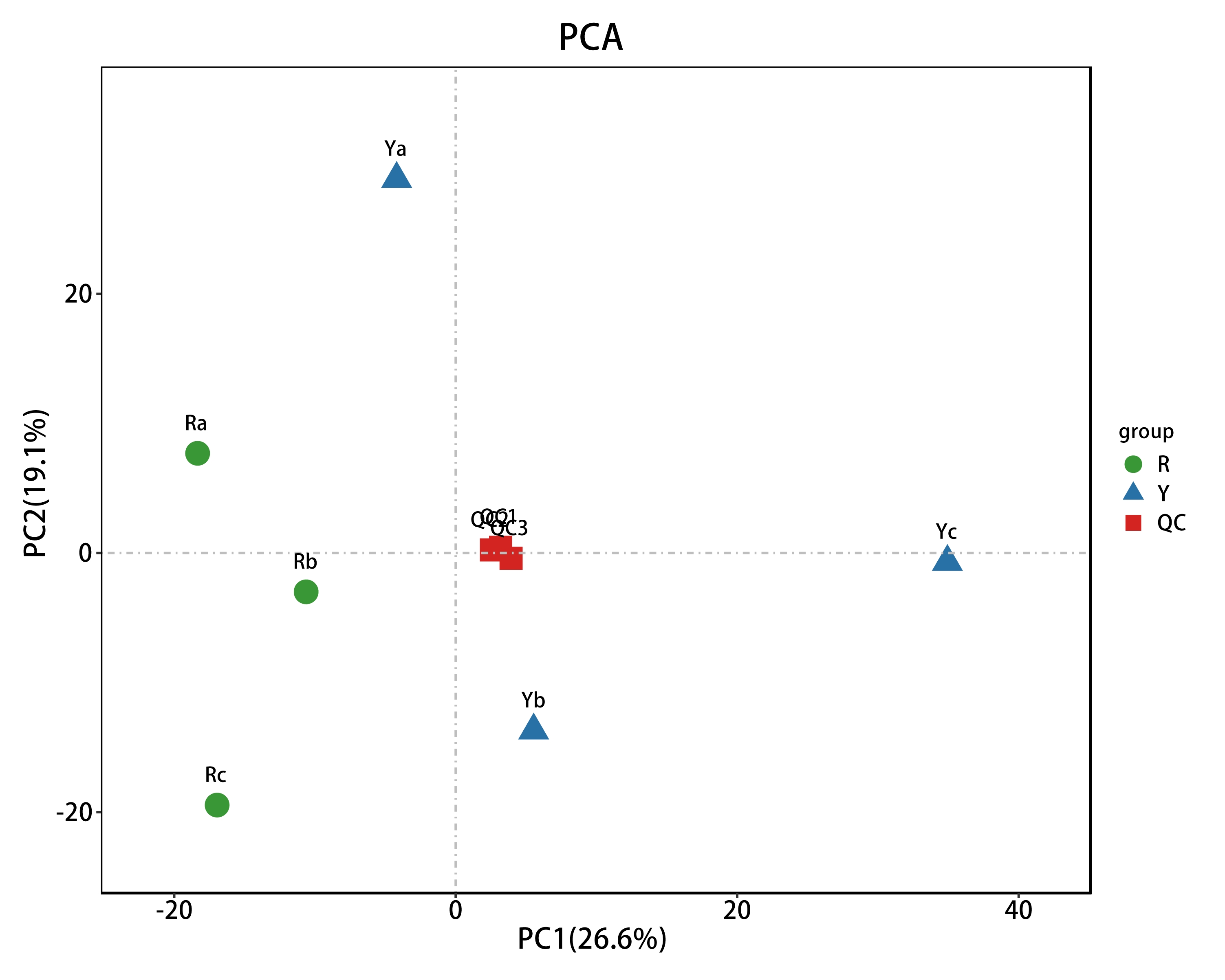


**Fig. S5** PCA plot of metabolites

**
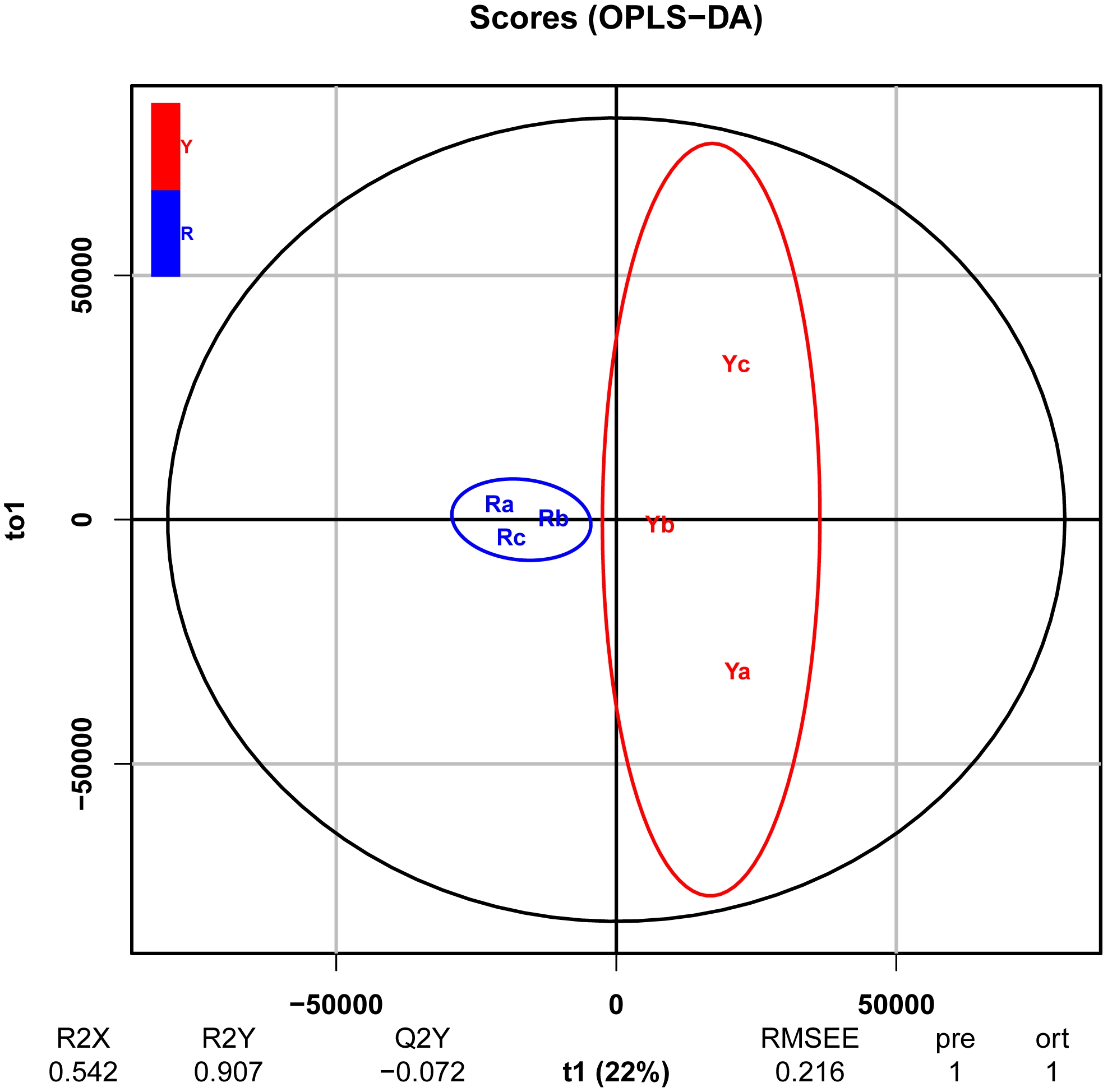
**

**Fig. S6** OPLS-DA of R VS Y.


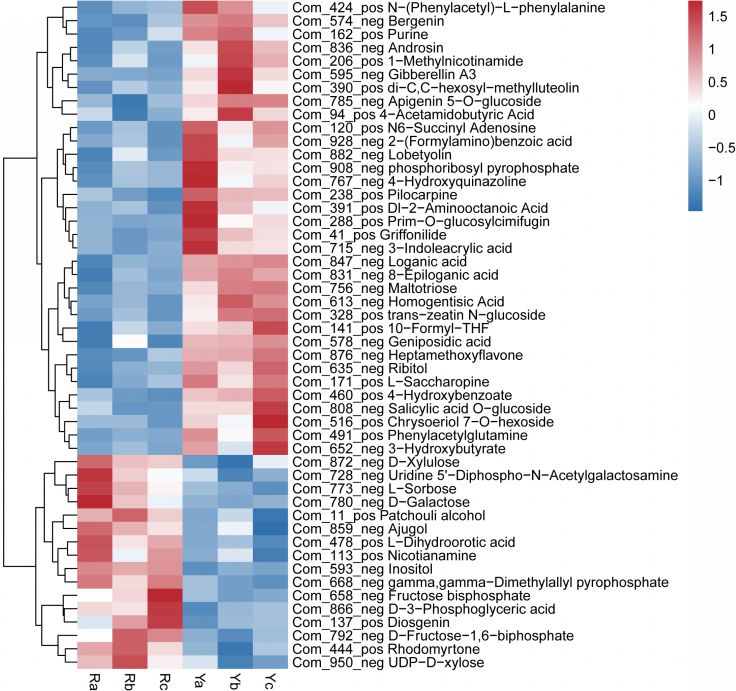


**Fig. S7** Cluster heat map of DAMs at R vs Y.

| 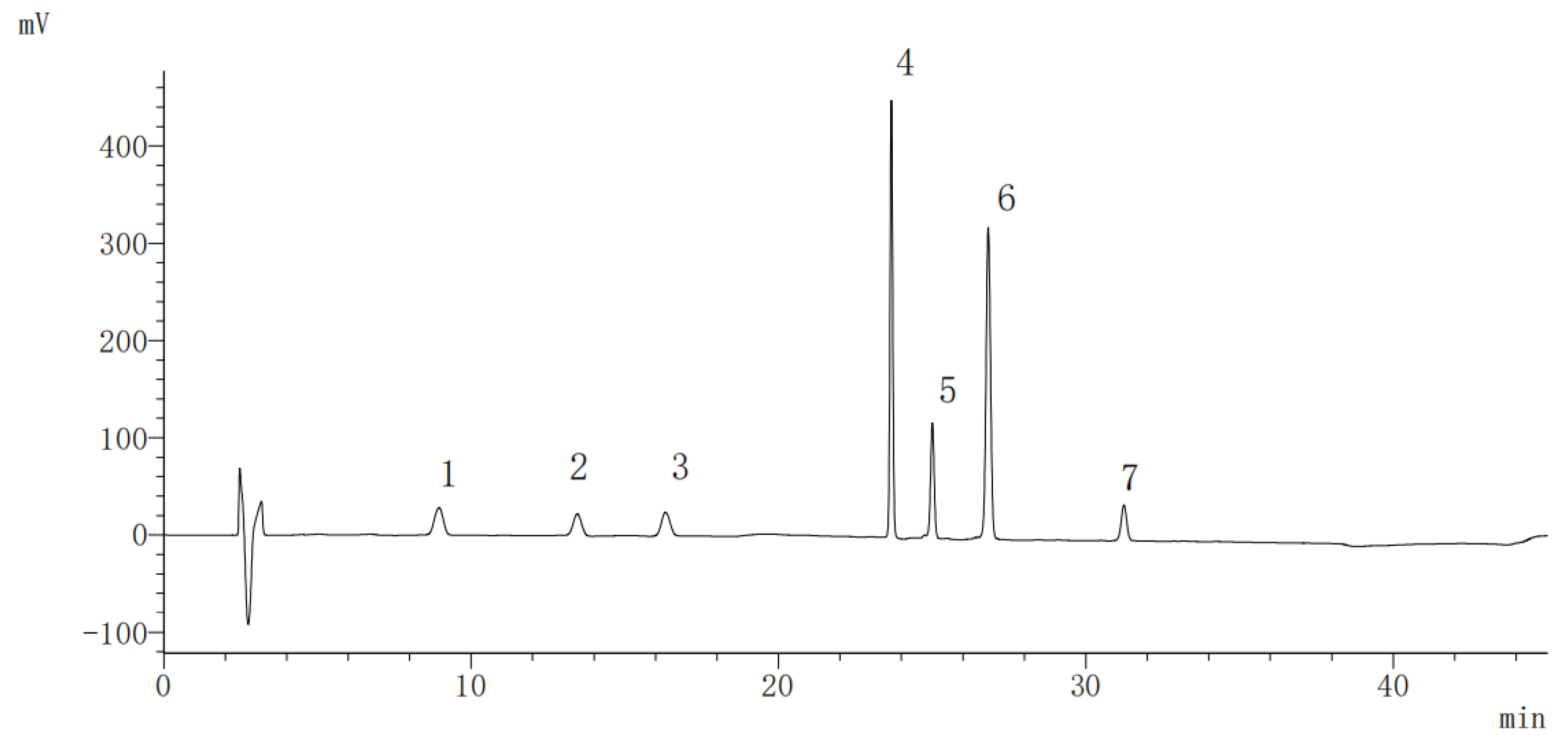 |
| --- |
| (A) |
| 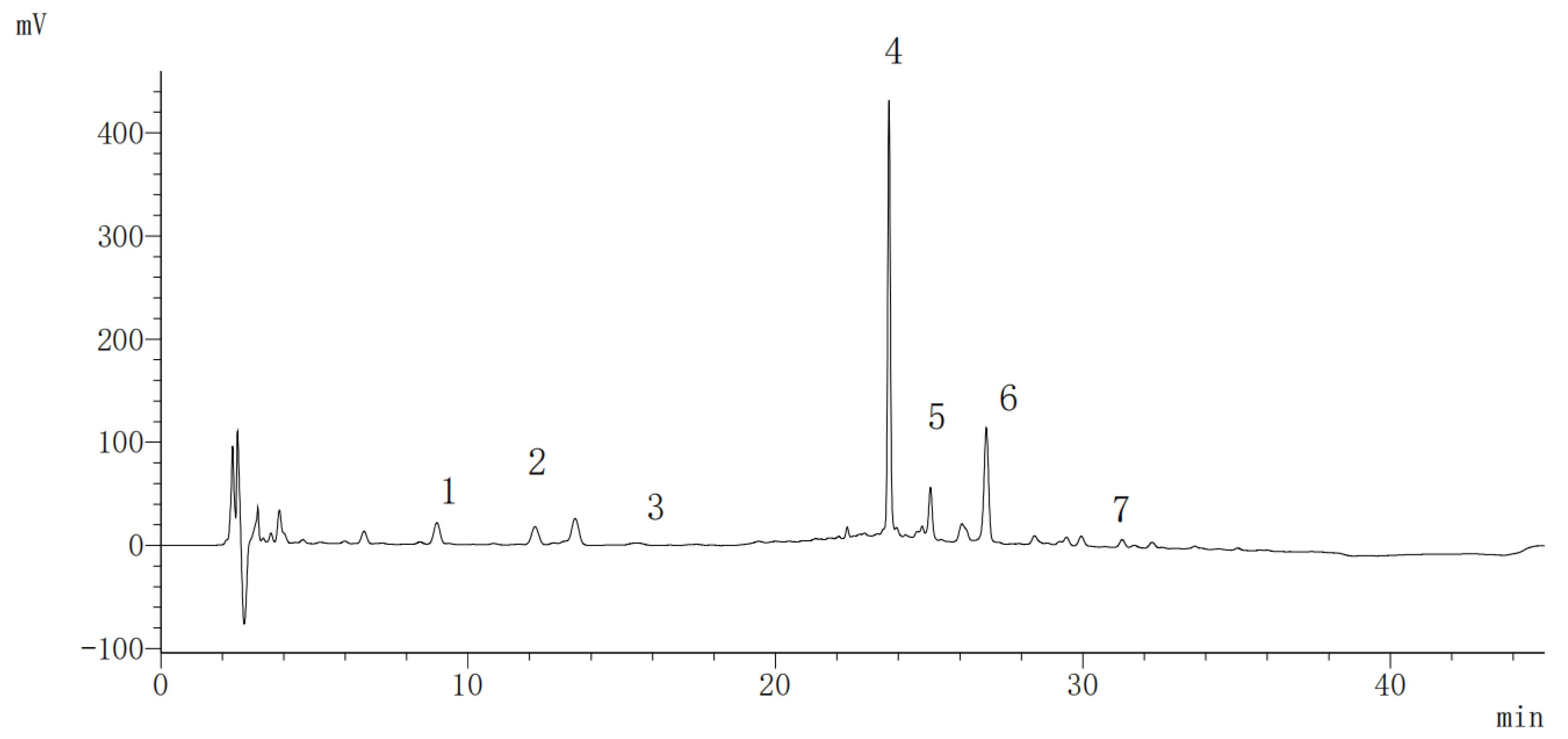 |
| (B) |
| 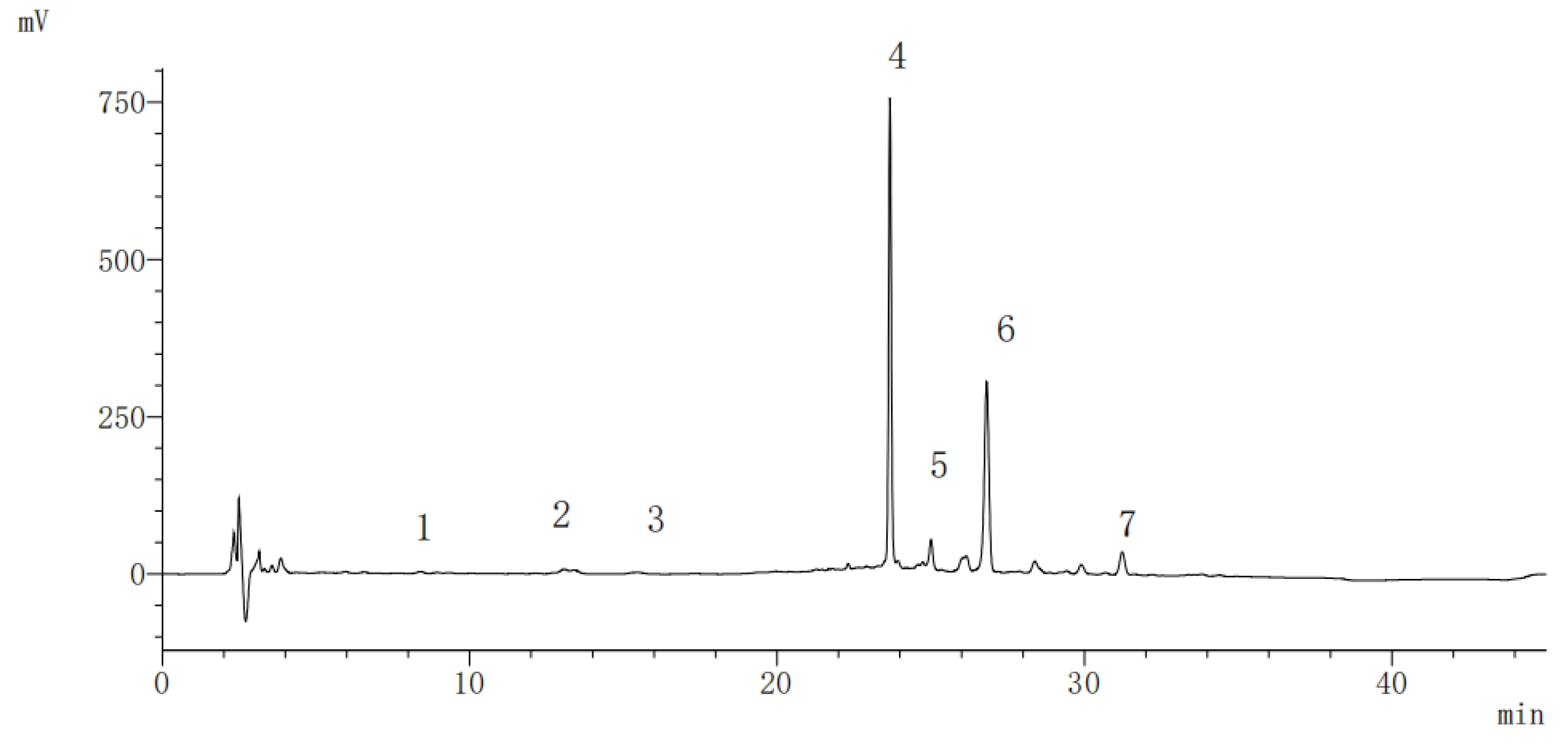 |
| (C) |

**Fig. S8** The high-performance liquid chromatogram of iridoids and phenylethanol glycosides. (1 for Geniposidic acid, 2 for 8-epiloganic acid, 3 for loganic acid, 4 for echicoside, 5 for cistancheside A, 6 for verbascoside, 7 for 2'-acetylacteoside)


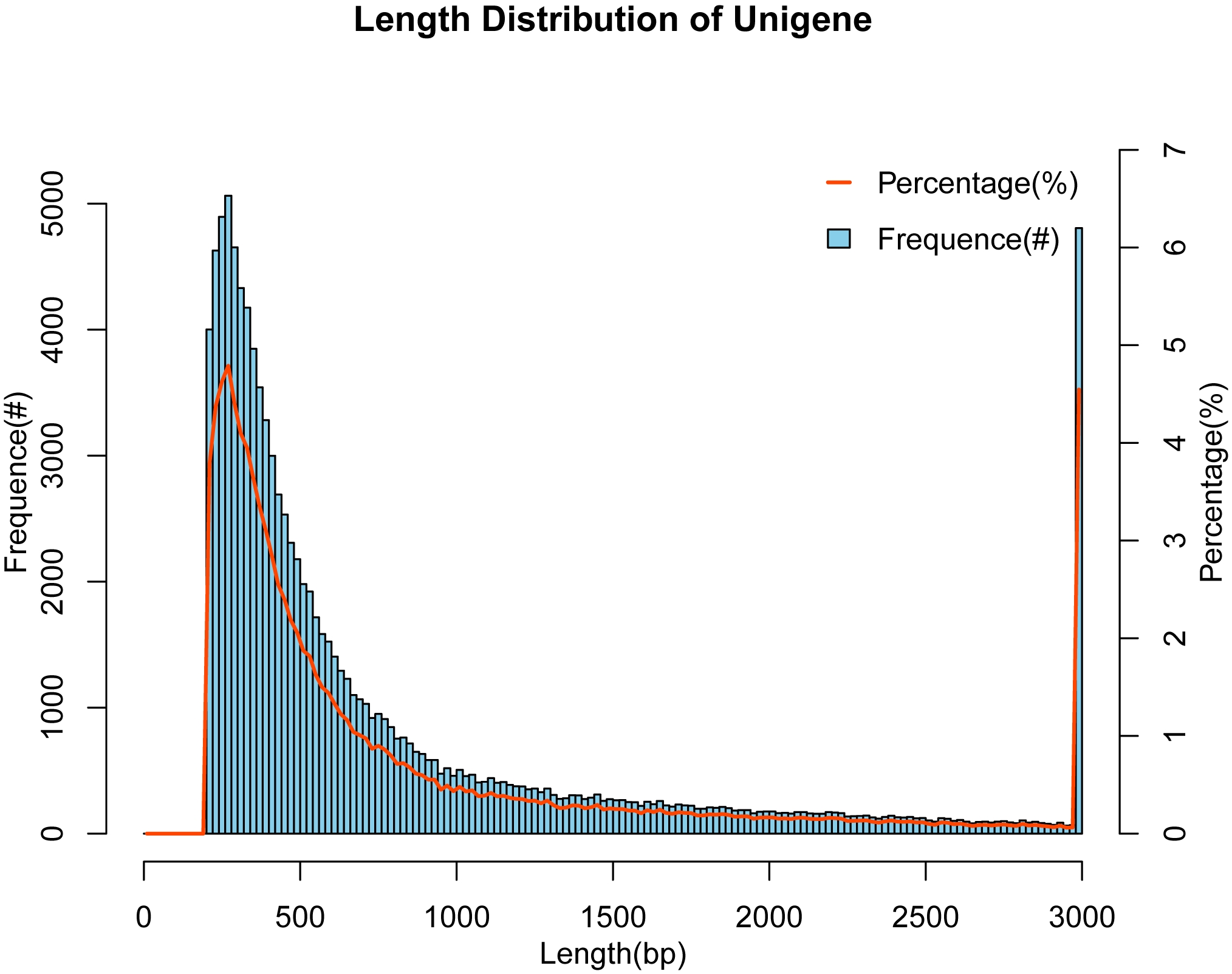


**Fig. S9** Unigene Length distribution plot.

**
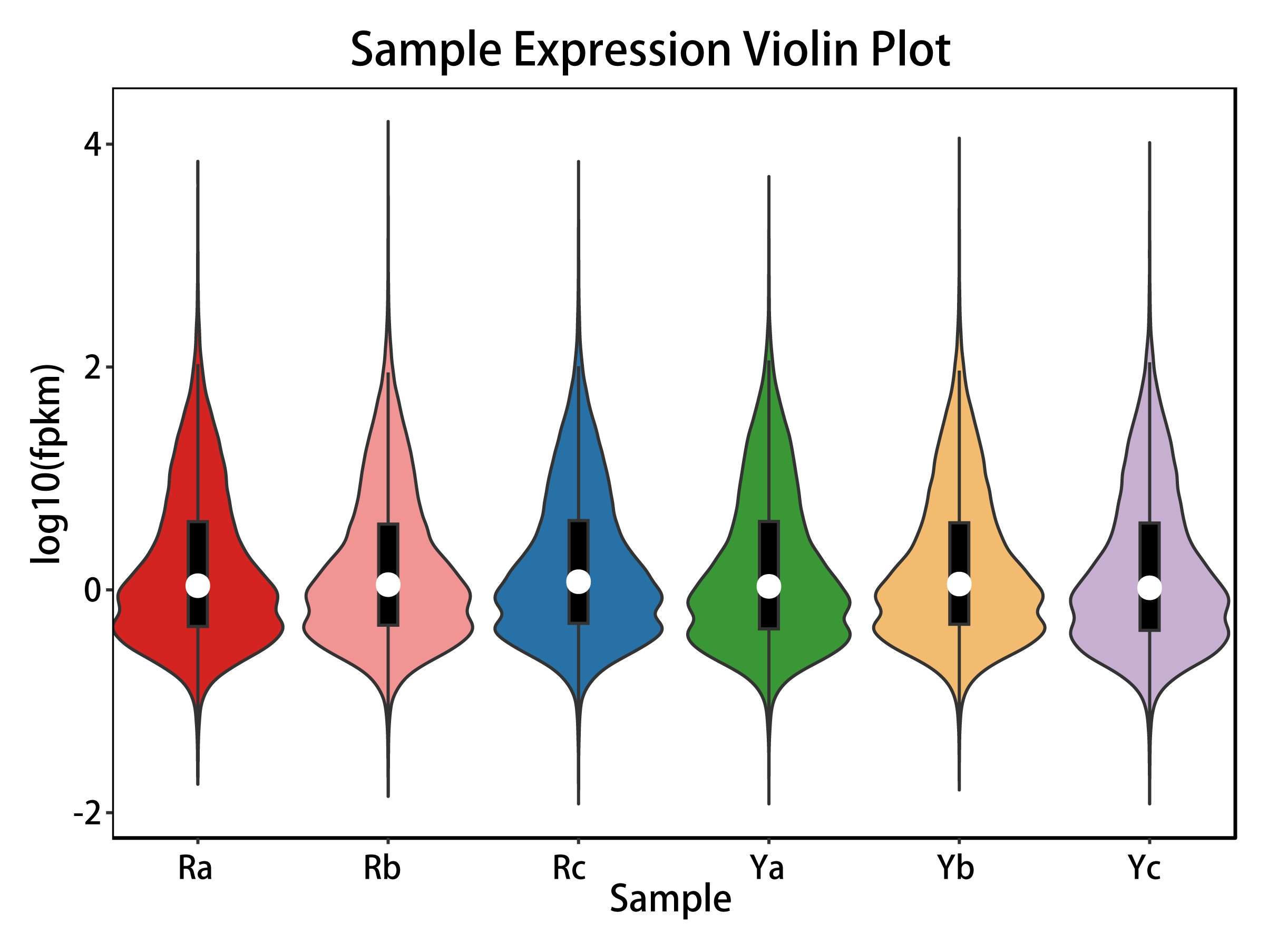
**

**Fig. S10** Violin plot of expression in R and Y.


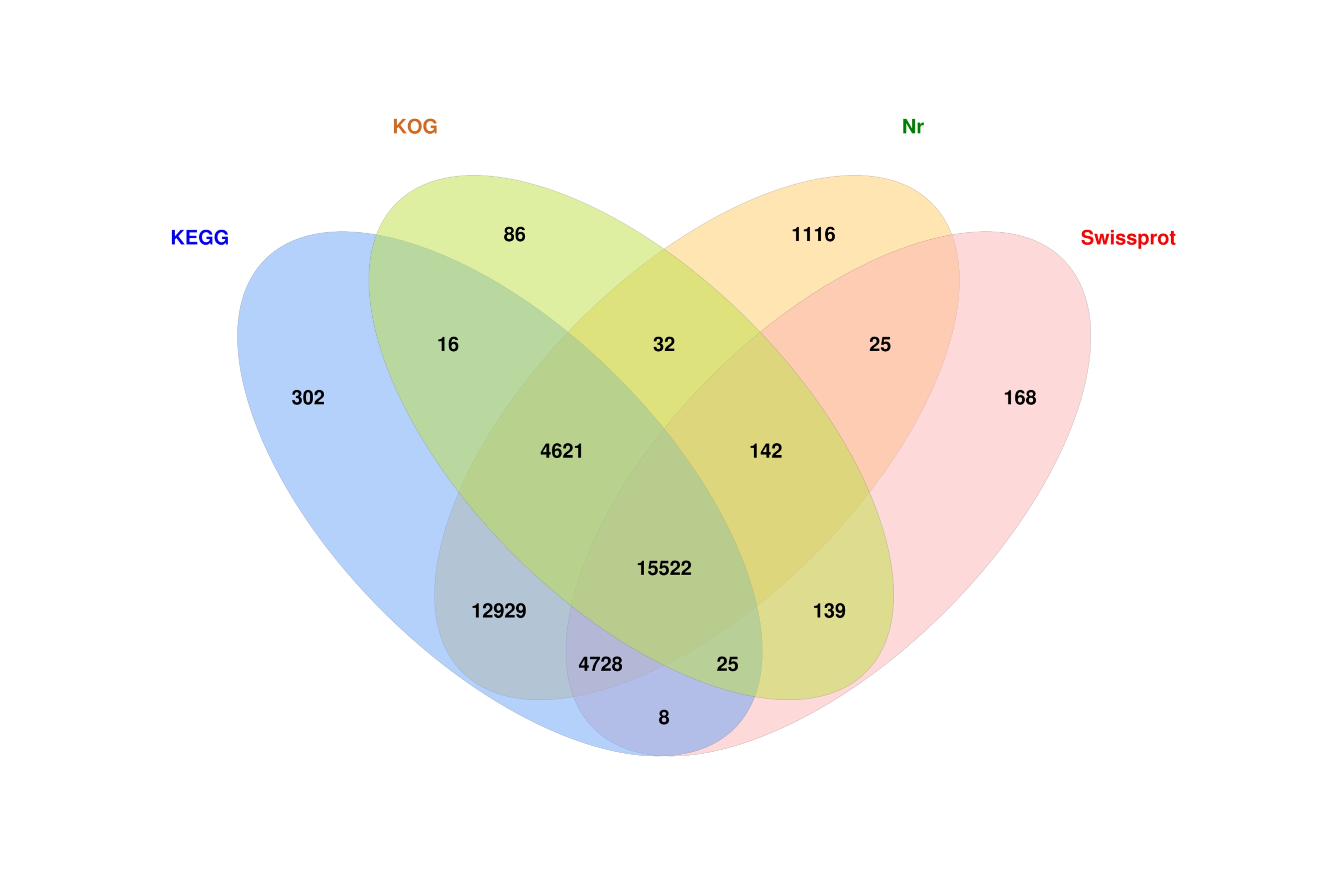


**Fig. S11** Venn diagrams determined using four major databases


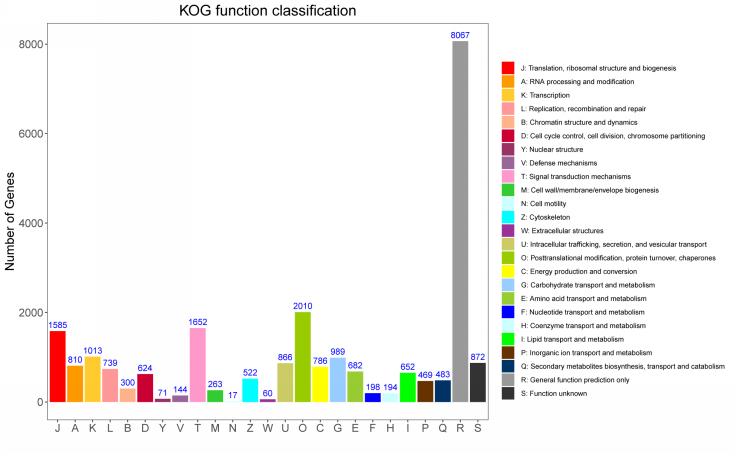


**Fig. S12** KOG function classification statistical plots.

**Table supplementary legends**

**Table S1 Specific primers of qRT-PCR amplification**

| Gene name | Primer sequence（5-3’） | Amplification length / bp |
| --- | --- | --- |
| *GAPDH* | F:GATGATGTTGAACTCGTCGC | 183 |
|  | R:CTCTTCTGGGTTTCTCACACC |  |
| *UGT85A24* | F: GCCGCCGCCTCATCAAGTC | 141 |
|  | R: TGGTGGTGGAGACGCAGAGC |  |
| *UGT9* | F: TAGACGAGTCGCAGATGGAGGAAG | 82 |
|  | R: TCCGAGGATCTTACCACCCACAAG |  |
| *4CLL1* | F: ACGCCAAAGAAAGTGAAGGAAGACG | 114 |
|  | R: CCCAGAAGGCGACTTTGGAATAGC |  |
| *6-FEH* | F: GGATGTTGGTCGGGTTCTGCTAC | 84 |
|  | R: GTTACCTTGCTCATCACCTCCTGTG |  |
| *AMY3* | F: CTTGTGAGGAGACTGCATGAAGTGG | 148 |
|  | R: CATCAGCAACAACTGCATGGTCATC |  |
| *F6H2-2-1* | F: ACTGGGAGGAAGAATAGGGTTTCGG | 113 |
|  | R: TGCTTGTAGATTGGCTTCTCACCTG |  |
| *GOLS2* | F: CCAGCCGCCGTCTCTTTACTTC | 104 |
|  | R: GAGTAGGAGGGGTGGTCTTGAGTG |  |
| *AGPS1* | F: TGCACCAGTTGATGACAGGTTTG | 129 |
|  | R: GCTGACGCTCGGCTGAGAAG |  |
| *CYP84A1* | F: TGGTGGAACGGAAACGGTAGC | 87 |
|  | R: TGCTTGACTCTCTTGAGGTCTTGTG |  |
| *SNL6* | F: TCACCAGCGGCGTATCCTAC | 150 |
|  | R: TGTTGTTGTTAAGTCTCATTTCACCAG |  |
| *FLS* | F: CCACCGTCTGCCATTGATTATCG | 149 |
|  | R: AGCTCGTCTGCCGCCAAC |  |
| *CCD4* | F: CACAGCAAGCATCGGAGACAG | 105 |
|  | R: CGACGGAGGGTTTACGAGGAG |  |
| *CCD7* | F: GCCATATTTCCCATTTGACACTGTTG | 95 |
|  | R: GCTCGCCTATGAATGTTCGTCTAC |  |

**Table S2** Differentially expressed genes of the metabolic pathways involved in this study.

| **Gene name** | **Protein name** | **log2(fc)** |
| --- | --- | --- |
| **Terpenoids synthesis pathway** | | |
| UGT85A24 | 7-deoxyloganetin glucosyltransferase | 1.554102324 |
| UGT87A2 | UDP-glycosyltransferase 87A2 | 2.141149972 |
| UGT9 | Beta-D-glucosyl crocetin beta-1,6-glucosyltransferase | 3.643009076 |
| GEAS | Germacrene A synthase | 3.079521546 |
| SS10 | Squalene synthase 10 | -4.324972827 |
| CCD4 | carotenoid cleavage dioxygenase 4 | -1.611207297 |
| CCD7 | carotenoid cleavage dioxygenase 7 | 1.965928135 |
| **Phenylpropanoid biosynthesis pathway** | | |
| IGS1 | Isoeugenol synthase 1 | 2.692010426 |
| CYP84A1 | Cytochrome P450 84A1 | 11.26150731 |
| 4CLL1 | 4-coumarate--CoA ligase-like 1 | -1.922333506 |
| F6H2-2-1 | Bi-functional coumaroyl CoA and feruloyl CoA ortho-hydroxylase F6H2-2-1 | -4.987803453 |
| EMB3004 | Bifunctional 3-dehydroquinate dehydratase/shikimate dehydrogenase | 4.882509423 |
| FLS | Flavonol synthase/flavanone 3-hydroxylase | -10.4005237 |
| 5MAT1 | Malonyl-coenzyme:anthocyanin 5-O-glucoside-6'''-O-malonyltransferase | -3.837297237 |
| SNL6 | Cinnamoyl-CoA reductase-like SNL6 | -1.701707564 |
| **Starch and sucrose metabolism pathways** | | |
| TPPJ | Probable trehalose-phosphate phosphatase J | 2.647195519 |
| RSS3 | Sucrose synthase 3 | -1.536241198 |
| SS3 | Starch synthase 3 | 6.377157615 |
| AMY3 | Alpha-amylase 3 | -10.45018025 |
| AGPS1 | Glucose-1-phosphate adenylyltransferase large subunit | 2.842011991 |
| **Galactose metabolic pathways** | | |
| 6-FEH | Fructan 6-exohydrolase | 1.650096332 |
| GOLS2 | Galactinol synthase 2 | -2.661226826 |

**Table S3 Determination table of the appearance and component content of oil cistanche and cistanche.**

| Composition/sample | | Oil cistanche | | | Average value | cistanche | | | Average value |
| --- | --- | --- | --- | --- | --- | --- | --- | --- | --- |
| X-L* | | 62.93 | 65.99 | 65.26 | 64.73 | 72.42 | 72.54 | 72.29 | 72.42 |
| X-a* | | 1.77 | 2.12 | 3.13 | 2.34 | 1.56 | 2.48 | 2.13 | 2.06 |
| X-b* | | 4.02 | 6.30 | 6.49 | 5.60 | 10.55 | 10.62 | 9.89 | 10.35 |
| G-L* | | 65.10 | 63.59 | 63.78 | 64.15 | 63.20 | 69.02 | 65.86 | 66.02 |
| G-a* | | 11.91 | 11.26 | 12.78 | 11.99 | 12.86 | 12.75 | 12.07 | 12.56 |
| G-b* | | 17.74 | 18.61 | 22.19 | 19.51 | 23.77 | 25.28 | 23.66 | 24.23 |
| Content  (mg/g) | Geniposidic acid | 0.71 | 0.68 | 0.80 | 0.73 | 0.07 | 0.05 | 0.06 | 0.06 |
|  | 8-Epi-Loganic acid | 2.96 | 3.47 | 4.05 | 3.49 | 0.79 | 0.73 | 0.71 | 0.74 |
|  | Loganic acid | 0.04 | 0.04 | 0.11 | 0.06 | 0.03 | 0.02 | 0.03 | 0.03 |
|  | polysaccharide | 1.89 | 2.46 | 1.83 | 2.06 | 1.18 | 1.25 | 1.33 | 1.25 |

| Components | Linear regression equation | R^2^ |
| --- | --- | --- |
| geniposidic acid | y=2.33974*10^7^x+6414.65 | 0.999 |
| 8-epiloganic acid | y=7.39731*10^6^x+8939.24 | 0.999 |
| loganic acid | y=2.68636*10^7^x-11676.5 | 0.999 |
| rutin | y=0.2856x-0.012 | 0.999 |
| anhydrous glucose | y=0.1063x-0.0158 | 0.999 |

**Table S4** Linear regression equation for the mixed control

Note: In the Linear regression equation of rutin and anhydrous glucose, x is the absorbance, and y is the concentration.
